# Cotton Bollworm (*H. armigera*) Effector PPI5 Targets FKBP17‐2 to Inhibit ER Immunity and JA/SA Responses, Enhancing Insect Feeding

**DOI:** 10.1002/advs.202407826

**Published:** 2024-10-01

**Authors:** Yaxin Wang, Chuanying Zhu, Gefei Chen, Xuke Li, Mingjv Zhu, Muna Alariqi, Amjad Hussian, Weihua Ma, Keith Lindsey, Xianlong Zhang, Xinhui Nie, Shuangxia Jin

**Affiliations:** ^1^ Hubei Hongshan Laboratory National Key Laboratory of Crop Genetic Improvement Huazhong Agricultural University Wuhan Hubei 430070 P. R. China; ^2^ Department of Biosciences Durham University Durham DH1 3LE UK; ^3^ Key Laboratory of Oasis Ecology Agricultural of Xinjiang Production and Construction Corps Agricultural College Shihezi University Shihezi Xinjiang 832003 P. R. China

**Keywords:** CRISPR/Cas9, cotton bollworms, cyclophilin, effectors, plant host immunity, endoplasmic reticulum

## Abstract

The cotton bollworm causes severe mechanical damage to plants during feeding and leaves oral secretions (OSs) at the mechanical wounds. The role these OSs play in the invasion of plants is still largely unknown. Here, a novel *H. armigera* effector peptidyl prolyl trans‐isomerase 5 (PPI5) was isolated and characterized. PPI5 induces the programmed cell death (PCD) due to the unfolded protein response (UPR) in tobacco leaf. We reveal that PPI5 is important for the growth and development of cotton bollworm on plants, as it renders plants more susceptible to feeding. The GhFKBP17‐2, was identified as a host target for PPI5 with peptidyl‐prolyl isomerase (PPIase) activity. CRISPR/Cas9 knock‐out cotton mutant (*CR‐GhFKBP17‐1/3*), VIGS (*TRV: GhFKBP17‐2*) and overexpression lines (*OE‐GhFKBP17‐1/3*) were created and the data indicate that *GhFKBP17‐2* positively regulates endoplasmic reticulum (ER) stress‐mediated plant immunity in response to cotton bollworm infestation. We further confirm that PPI5 represses JA and SA levels by downregulating the expression of JA‐ and SA‐associated genes, including *JAZ3/9*, *MYC2/3*, *JAR4*, *PR4*, *LSD1*, *PAD4*, *ICS1* and *PR1/5*. Taken together, our results reveal that PPI5 reduces plant defense responses and makes plants more susceptible to cotton bollworm infection by targeting and suppressing *GhFKBP17‐2* ‐mediated plant immunity.

## Graphical abstract

The *H. armigera* effector PPI5 is secreted into plant cells where it targets FKBP17‐2, inhibiting ER immunity and JA/SA responses. This allows the cotton bollworm to boost feeding and development.

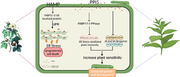

## 1 Introduction

Plant‐insect interaction is considered as one of the most ancient and co‐evolved systems, as many insects feed on plant pollen, nectar, and leaves and many plants reproduce through insect pollination. During this long period of co‐evolution, plants and insects have developed molecular mechanisms to detect each other's defenses.^[^ ^1^, ^2^, ^3^, ^4^, ^5^ ^]^ Unable to move away from insect predators, plants have evolved adaptations to improve their reproduction and survival, such as epidermal trichomes, leaf patterns, and waxes to resist insect feeding, and they also produce toxins as deterrents. Some plants secrete signal molecules to attract insect predators to defend themselves against insects.^[^ ^6^, ^7^ ^]^ Some insects have developed unique mouthparts that can feed on plants, ensuring that they acquire enough nutrients to reproduce. They even inhibit plant defenses by secreting saliva, feces, and pheromones which are known as effectors, while other compounds known as elicitors can trigger plant defense responses.^[^ ^8^, ^9^, ^10^ ^]^

Most effector studies have been conducted on sap‐sucking insects such as aphids: Me10, Me23,^[^ ^11^, ^12^ ^]^ Mp10, Mp42,^[^ ^13^ ^]^ Armet,^[^ ^14^ ^]^ Sm9723,^[^ ^15^ ^]^ Sg2204^[^ ^16^ ^]^; whiteflies: Bt56,^[^ ^17^ ^]^ Armet,^[^ ^18^ ^]^ Bsp9,^[^ ^19^ ^]^ LsPDI1,^[^ ^20^ ^]^ LsSP1^[^ ^21^ ^]^; brown planthoppers NlEG1,^[^ ^22^ ^]^ NlSEF1,^[^ ^23^ ^]^ Vg,^[^ ^24^ ^]^ DNase II^[^ ^25^ ^]^; nematodes: Pp‐EXPB1,^[^ ^26^ ^]^ MeTCTP^[^ ^27^ ^]^ and MjTTL5.^[^ ^28^ ^]^ The effectors inhibit callose deposition, sieve tube blockage, and plant cell wall degradation by suppressing the accumulation of Ca^2+^, hydrogen peroxide, and ROS bursts. They also affect other plant defense responses such as disrupting the crosstalk between, and accumulation of, SA and JA, decreasing plant proteasome activity, targeting other defense‐related proteins, triggering pathogen responses, and interfering with the signal transduction pathway to promote plant susceptibility and insect feeding. The elicitors induce plant immune responses by inducing ROS bursts, salicylic acid, and H_2_O_2_ accumulation, defense gene expression and hypersensitive responses, plasma membrane calcium influx, and membrane depolarization; such elicitors include spidermite TePDI,^[^ ^29^ ^]^ aphid CathB3,^[^ ^30^ ^]^ beet armyworm FAC,^[^ ^9^ ^]^ *Spodoptera exigua* inceptins,^[^ ^31^ ^]^ *Pieris brassicae* eggs phosphatidylcholines,^[^ ^8^ ^]^ *Tetranychus urticaes* Tet1, Tet2.^[^ ^32^ ^]^ The first reported effector produced by chewing insects was the enzyme glucose oxidase (GOX) from cotton bollworm (*Helicoverpa armigera*), which inhibited nicotine production in tobacco but induced a defense response in tomatoes.^[^ ^33^, ^34^ ^]^ Two other effectors, HARP1 and HAS1 from *H. armigera*, were found to suppress JA defense responses.^[^ ^35^, ^36^, ^37^ ^]^ Most research in insect effectors has focused on sap‐sucking insect effectors, while research on chewing insect effectors and their mechanisms to modulate plant defense responses are largely unknown.

Diverse molecular processes regulate the interaction between plants and insect herbivores. Plants recognize HAMPs (herbivore‐associated molecular patterns) and adjust their defense responses against insect herbivores.^[^ ^29^, ^38^, ^39^, ^40^ ^]^ The plant immune responses consist of pattern‐triggered immunity (PTI) and effector‐triggered immunity (ETI).^[^ ^41^ ^]^ PTI and ETI often rely on the ER quality control system^[^ ^42^ ^]^ and hormone signaling.^[^ ^43^ ^]^ The ER is a membrane‐bound compartment that mediates cellular processes.^[^ ^44^ ^]^ Secreted proteins are translocated into the ER and are properly folded and modified to guarantee their functionality before being transported to their final destination.^[^ ^45^ ^]^ Under abiotic or biotic stress, unfolded or misfolded proteins often accumulate in the ER lumen, which results in ER stress. To relieve ER stress and restore ER homeostasis, ER membrane‐localized stress sensors subsequently activate the UPR.^[^ ^46^ ^]^ The UPR includes the induction of ER chaperones and foldases, such as heat‐shock proteins (HSPs), protein disulfide isomerases, and PPIases, which enhance protein folding.^[^ ^47^ ^]^ In addition, the efficiency of protein translation is attenuated, global gene expression is inhibited, the capacity of protein secretion is potentiated and ER‐associated protein degradation is induced in order to restore ER homeostasis and hence functionality.^[^ ^43^, ^45^ ^]^ Conditions of chronic or irreversible ER stress trigger cell death by apoptosis.^[^ ^46^, ^48^, ^49^ ^]^ However, the molecular mechanisms of how ER‐associated or ‐regulated processes participate in plant immunity during plant–insect interactions are not well investigated yet.

The cyclophilins (CYPs) of *Magnaporthe grisea*, *Botrytis cinerea*, and *Cryphonectria parasitica* are important for infection of host plants, as *Cyp* mutations led to reduced virulence and impaired function.^[^ ^50^, ^51^, ^52^ ^]^ In animals, *Toxoplasma gondii* CYP18 mediates anti‐inflammatory signaling in the host cell to maximize parasite replication and transmission while maintaining host survival.^[^ ^53^ ^]^ *Drosophila* shutdown (shu), which contains domains involved in peptidyl‐prolyl cis‐trans isomerase (PPI) activity and binding of HSP90 family chaperones, plays an important role in primary biogenesis and adaptive amplification cycles.^[^ ^54^ ^]^ *Trypanosoma cruzi* secretes PPI that binds and neutralizes the reduced antimicrobial peptide test lysin, thereby promoting parasite survival.^[^ ^55^ ^]^ *Apolygus lucorum* Al106 is also a PPI that inhibits plant immunity and promotes insect feeding by interacting with plant PUB33.^[^ ^56^ ^]^ *Spodoptera exigua* PPIs have been identified in the labial salivary glands of caterpillars feeding on a nutritionally poor diet.^[^ ^57^ ^]^ All of the above studies indicate that CYPs from a variety of organisms are important for the virulence of infestation and adaptive metabolic responses.^[^ ^55^ ^]^ However, the function of CYPs in *H. armigera* is unknown.

The immunophilin protein family has been involved in a variety of cellular activities and responses to a wide range of biotic and abiotic stimuli. They are among the most highly conserved proteins in eukaryotes and prokaryotes.^[^ ^58^ ^]^ Since the advance of genome sequencing technologies, individuals of this gene family have been widely discovered in yeast, worms, humans, *Arabidopsis*, rice, and Chlamydomonas.^[^ ^59^, ^60^, ^61^, ^62^, ^63^ ^]^ They play multiple roles in hormone response, transcriptional regulation, protein folding, signal transduction, abiotic stress, and stress defense responses.^[^ ^64^, ^65^, ^66^, ^67^, ^68^ ^]^.

Plant immunophilins were previously shown to be involved in the function of innate immunity in higher plants.^[^ ^69^ ^]^ Immunophilins are a family of enzymes with a PPIase activity.^[^ ^70^ ^]^ Two groups of immunophilin receptors exist in plants: cyclosporin A receptors, often referred to as *CYPs*, and the *FK506‐* and *rapamycin‐binding proteins* (*FKBPs*).^[^ ^71^ ^]^ *At5g48570*, which is also known as *ROF2* (*AtFKBP65*), encodes a heat stress protein that participates in long‐term acquired thermotolerance. *Arabidopsis AtFKBP65* knockout mutations result in increased susceptibility to *P. syringae*, whereas overexpression of *AtFKBP65* alters the transcriptional profile of pathogen‐related defense genes and leads to enhanced resistance.^[^ ^71^ ^]^ *Polytrichastrum alpinum* peptidyl‐prolyl isomerase *FKBP12* (*PaFKBP12*) expression is induced by heat and ABA. Overexpression of *PaFKBP12* in *Arabidopsis* confers stress tolerance to heat stress, ABA, drought, and salinity.^[^ ^72^ ^]^ The closest Arabidopsis CPR1 homolog, ROC1 has PPIase cyclophilin activity that activates *Pseudomonas syringae* type III virulence effector AvrRpt2, enabling it to cleave its N terminus, localize to the membrane where RPS2 and RIN4 reside, and directly cleave RIN4. Perturbation of RIN4 activates RPS2‐mediated resistance.^[^ ^73^, ^74^, ^75^ ^]^ Transcriptional activator‐like effector of the citrus canker pathogen *Xanthomonas citri*, named PthA2, targets the cyclophilin CsCyp. PthA2 inhibits the PPIase activity of CsCyp, and the silencing of CsCyp enhances canker lesions in *X. citri*‐infected leaves.^[^ ^76^ ^]^ The Phytophthora effector Avr3b directly interacts with the soybean cyclophilin GmCYP1, which activates the hydrolase activity of Avr3b in a PPIase activity‐dependent manner.^[^ ^77^ ^]^ *Phytophthora capsici* RXLR effector, PcAvr3a12, facilitates infection by targeting and suppressing a novel ER‐localized PPIase, FKBP15‐2, which is required for ER stress‐mediated plant immunity.^[^ ^43^ ^]^ The *Bursaphelenchus xylophilus* effector BxML1 targets a CYP to promote parasitism and virulence in pine.^[^ ^78^ ^]^

*H. armigera* is one of the most destructive insect pests in the world, causing severe damage to cotton, maize, vegetables, and fruit trees. In this study, we identified an *H. armigera* effector, PPI5 (*XP_021198138.1*), which regulates the defense response of cotton (*Gossypium hirsutum*) and tobacco (*Nicotiana tabacum*). PPI5 targets the cotton ER‐localized GhFKBP17‐2 and inhibits ER stress‐mediated plant immunity and JA defense response by suppressing *GhFKBP17‐2* transcription and the functional enzymatic activity of proline cis‐trans isomerase, and inhibits both the JA and SA responses in tobacco. This results in plants being more susceptible to cotton bollworm invasion, allowing better growth and development of the cotton bollworm on the host plants.

## 2 Results

### 2.1 Identification of PPI5 in Cotton Bollworm

In order to identify new effectors, we collected the OSs of *H. armigera* fed on cotton leaves and performed a qualitative proteomic analysis. A total of 233 proteins were identified (Figure S1, Supporting Information). After the prediction of effectors by EffectorP, 47 candidate proteins were analyzed further. Among these proteins, 2 with predicted transmembrane domains were ruled out considering that they were likely to be anchored to membranes. Of the remaining 45 proteins, 17 were digestion‐related. We amplified the remaining 28 proteins from an *H. armigera* cDNA library, which allowed us to obtain 19 candidate effector genes. After sequencing, 9 genes with correct amino acid sequences were confirmed based on predictions (Figure S2A,B, Supporting Information).

**Figure 1:**
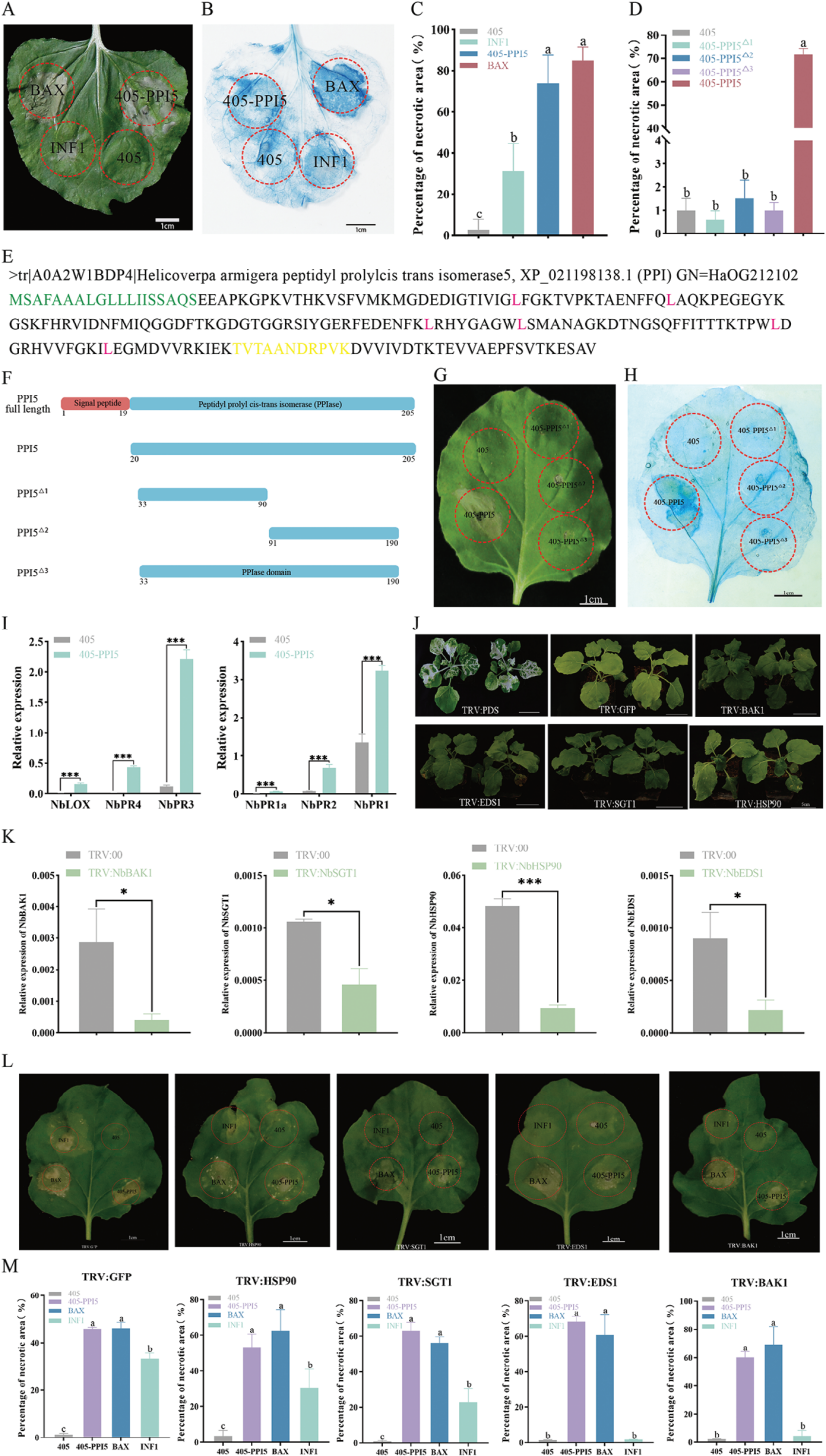
The PPI5 induces programmed cell death to *N. benthamiana*. A–C) Transient expression of the empty vector pGWB405 (405), 405‐PPI5, positive control INF1 and BAX. 405‐PPI5 induced cell death in *N. benthamiana*. Infected leaves were stained with Trypan blue at 48 h to visualize necrotic areas. Error bar, ±SD (*n  =*  6 biological replicates). Significance was examined by one‐way ANOVA, *p<*0.05. Scale bars, 1 cm. E) The amino acid (AA) sequence of PPI5. Signal peptide (1–19 AA, green font) that is predicted by SignalP‐5.0. The unique peptide identified from LC–MS/MS (yellow font). The amino acid sequence of PPI5 contains six leucines (red font). F) Schematic diagram showing the protein structures of PPI5 and its deletion mutants. Structural domains of PPI5 as analyzed by the National Library of Medicine (NCBI) and UniProt. The red part is the signal peptide and the blue part is the structural domain of peptidyl‐prolyl cis‐trans isomerase of PPI5. Numbers underneath each construct indicate amino acid positions. D,G,H) 405‐PPI5 mutants lost the ability to induce cell death in *N. benthamiana*. The pictures were taken at 48 h after transient expression of the 405, 405‐PPI5, and 405‐PPI5 mutants in *N. benthamiana*. Infected leaves were stained with Trypan blue at 48 h to visualize necrotic areas. Error bar, ±SD (*n  =*  6 biological replicates). Significance was examined by one‐way ANOVA, *p <*0.05. Scale bars, 1 cm. I) Relative expression of plant defense‐related marker genes in *N. benthamiana* leaves. Leaves were collected at 48 h after *Agrobacterium tumefaciens* infestation. J) Symptoms of *N. benthamiana* when its gene was silenced by VIGS. Scale bars, 5 cm. VIGS assays were conducted at the 4‐leaf stage of *N. benthamiana*. Symptoms of *N. benthamiana* after silencing were photographed after 5 weeks of growth. Phytoene desaturase (*PDS*) was used for a positive control. GFP was used as a negative control. K) Silencing efficiency of *SGT1*, *HSP90*, *EDS1* (enhanced disease susceptibility1), and *BAK1* (BRI1‐associated kinase1), in the indicated gene knock‐down *N. benthamiana* plants. Error bar, ±SD (*n  =*  3 biological replicates). Asterisks represent statistically significant differences from Student's t‐tests (*p < 0.05, **p < 0.01, ***p < 0.001). L,M) Transient expression of PPI5 in PTI‐ and ETI‐related gene silencing *N. benthamiana* leaves. 405, BAX, and INF1 were used as the negative control and positive control, respectively. Scale bars, 1 cm. Error bar, ±SD (*n  =*  6 biological replicates). Significance was examined by one‐way ANOVA, *p <* 0.05.

Transient expression in *N. benthamiana* leaf was used to screen potential effector proteins of *H. armigera*. Nine candidate genes were constructed in the pGWB405 (405) *Agrobacterium* vector and injected into *N. benthamiana* to detect to test if cell death occurs.^[^ ^24^, ^29^ ^]^ In the cell death induction assay, 405 empty vectors were used as a negative control, and PAMPs (pathogen‐associated molecular patterns) INF1 and BAX, which are the most commonly used cell death inducers in plant immunity studies, were used as positive controls. One of the candidate genes, *PPI5*, induced cell death in *N. benthamiana* leaf cells. Compared to the positive controls, BAX and INF1, PPI5 caused significantly greater necrosis than the empty vector 405, which was consistent with the area of necrosis for *BAX* (**Figure** **1**A–C).

Studies on plant pathogens and insects revealed some common features of effector proteins such as short amino acid sequences, cysteine‐rich residues, or high sequence diversity.^[^ ^79^, ^80^ ^]^ *PPI5* encodes a peptidyl‐prolylcis‐trans isomerase 5, which contains 205 amino acids with a signal peptide of 19 amino acids. The qualitative proteomic assay identified a unique peptide, 11 AA (TVTAANDRPVK), which matched PPI5. PPI5 contains six prolines, which is consistent with the basic characteristics of secreted proteins (Figure 1E).

To identify functional domains of PPI5, we characterized potential domains using the database from the National Library of Medicine (https://www.ncbi.nlm.nih.gov/cdd) and UniProt (https://www.uniprot.org/). A functional domain of PPIase was identified (Figure 1F) and three deletion mutants were generated to test its function: PPI5^△1^ (retention of amino acids 33–90); PPI5^△2^ (retention of amino acids 91–190); and PPI5^△3^ (retention of amino acids 33–190) (Figure 1F). These mutants were transiently expressed in *N. benthamiana*. *PPI5* mutants lose capacity for cell death induction (Figure 1G,H). These results suggest that the PPIase domain is essential for the function of *PPI5*. (Figure 1A–H).

In addition, the expression levels of JA‐related genes LOX (lipoxygenase), PR3 (pathogenesis‐related protein 3) and PR4 (pathogenesis‐related protein 4) and SA (salicylic acid)‐related genes NPR1 (nonexpressor of pathogenesis‐related genes 1), NPR2 and PR1a (pathogenesis‐related protein 1a) were significantly upregulated in leaves induced by 405‐PPI5 relative to control 405 (Figure 1I). This indicates that PPI5 is a potent key protein that induces N. benthamiana defense responses. To determine whether PPI5‐mediated N. benthamiana cell death is recognized by PTI and ETI, we knocked down the expression of PTI and ETI‐regulated genes by virus‐induced gene silencing (VIGS) assay in *N. benthamiana* (Figure 1J,K). We found many PTI‐ and ETI‐regulating genes, such as BAK1, SGT1, HSP90, and EDS1, were also not required for PPI5‐mediated cell death (Figure 1L,M). Our results suggest that PPI5‐induced cell death is not dependent on pathways mediating cell death associated with PTI and ETI.

### 2.2 PPI5 is a Secreted Protein

**Figure 2:**
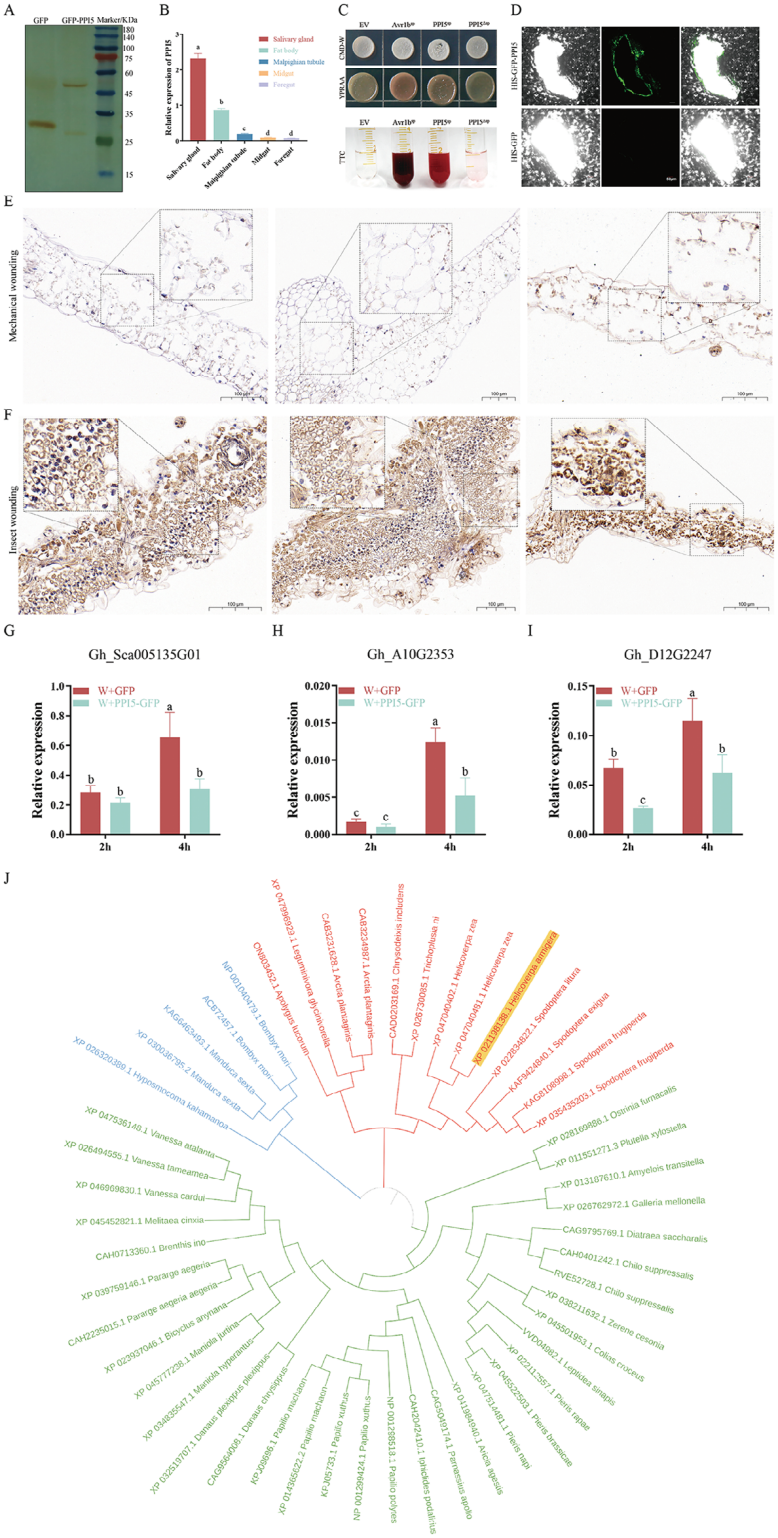
*H. armigera* oral protein PPI5 is characterized as a secreted protein. A) Western blot detection of PPI5 protein in plants using an anti‐GFP antibody. Tobacco plants were infested by *Agrobacterium tumefaciens* for 48 h. B) Transcription of *PPI5 in* various tissues and organs of *H. armigera*. C) Analysis of yeast signal peptide secretion in PPI5. D) Overview of HIS‐GFP‐PPI5 fusion protein fluorescence around *N. benthamiana* wounding site using confocal microscopy. Scale bars, 50 µm. E,F) Whole amount immunohistochemistry detection of PPI5 at the chewing sites of cotton leaves. The mechanical wounding leaves were used as a negative control. Anti‐PPI5 antibody was used to detect PPI5 in E‐F. Black dotted box for partial image enlargement. G–I) Expression of protease inhibitors in cotton. Fusion protein was applied around cotton mechanical damage. Samples were collected 2 and 4 h later, and the gene expressions were detected by qRT‐PCR. Error bar, ±SD (*n  =*  3 biological replicates). Significance was examined by one‐way ANOVA, *p <*0.05. J) Evolutionary tree analysis of PPI5.

Most reported effectors have a secretory function and can be secreted into plants to interfere with plant defense responses, and we then hypothesized that the identified PPI5 may be secreted and in contact with plants during feeding. When the GFP‐PPI5 fusion protein was transiently expressed in *N. benthamiana*, western blotting was performed to verify that the GFP‐PPI5 was secreted into plant cells (**Figure** **2**A). We then dissected the fourth instar cotton bollworm larvae and collected the salivary gland, fat body, midgut, foregut, and malpighian tubule for *PPI5* transcription analysis. The qRT‐PCR results showed that *PPI5* was significantly highly expressed in the salivary gland (Figure 2B). We then performed yeast signal peptide experiments. We constructed pSUC2 plasmids for the signal peptide of PPI5 (PPI5^sp^) and PPI5 without the signal peptide (PPI5^ΔSP^) and then transformed into an invertase drug‐deficient yeast strain YTK12. The previously published signal peptide of *Phytophthora sojae* effector Avr1b was used as the positive control.^[^ ^81^ ^]^ YTK12 with pSUC2 (empty vector, EV) was used as the negative control. PPI5^sp^ and the Avr1b^sp^ strains were able to grow normally on the YPRAA medium and discolored the TTC solution, whereas PPI^ΔSP^ and EV could not (Figure 2C). To visualize the PPI5 protein in plant tissues, PPI5 was fused to GFP, which was expressed in PET‐28‐a (Figure S3, Supporting Information), and the controls His‐GFP and His‐GFP‐PPI5 were applied to mechanically damaged *N. benthamiana* leaves. After 1 h, the fluorescence signal of His‐GFP‐PPI5 was detectable around the damage sites, but not that of His‐GFP (Figure 2D). In order to ascertain whether PPI5 can be secreted into plants via the feeding wound, whole‐amount immunohistochemistry was employed as a direct demonstration technique. Firstly, a polyclonal antibody against PPI5 was applied to detect the total protein of the cotton bollworm. The results of the Western blot (WB) demonstrated that the antibody was specific, with the band size matching the protein molecular weight of PPI5 (Figure S4A, Supporting Information). Then, we injected dsRNA into second instar larvae to interfere with the transcription of *PPI5* in cotton bollworm. The WB assay demonstrated that the antibody was unable to detect PPI5 bands 48 h after the administration of dsPPI5 (Figure S4A, Supporting Information). Consequently, WT and dsPPI5 bollworms were permitted to feed freely on cotton leaves, with mechanical damage serving as a control for immunohistochemistry. A whole amount of immunohistochemistry revealed that PPI5 was deposited at the larval injured sites of the cotton leaves during feeding (Figure 2F). Conversely, antibody signals could not be detected in mechanically damaged and dsPPI5 cotton bollworm‐fed cotton leaves. (Figure 2E; Figure S4B, Supporting Information). The expression of plant protease inhibitor genes can be induced by insect feeding and mechanical damage, which was considered to be an anti‐insect defense compound.^[^ ^82^ ^]^ We then used the cotton bollworm host plant, *G. hirsutum*, to detect the effects of PPI5 on plant defense. The recombinant His‐GFP‐PPI5 and His‐GFP proteins were applied to the cotton leaf injured sites. Cotton genes encoding protease inhibitors (*Gh_Sca005135G01*, *Gh_A10G2353*, and *Gh_D12G2247*) showed a rapid response to wounding, and their wound induction in leaves was significantly suppressed by PPI5 application (Figure 2G–I). The evolutionary relationships of 51 PPIs, including Al106, were compared based on their amino acid sequences, and the results showed that PPI5 is highly conserved in the nocturnal moth family Lepidoptera (Figure 2J). These results all suggest that PPI5 plays a conserved key role in the development of *H. armigera* and is secreted into the plant to interfere with plant defense responses.

### 2.3 Overexpression of *PPI5* in Cotton and Tobacco Promotes Bollworm Feeding

**Figure 3:**
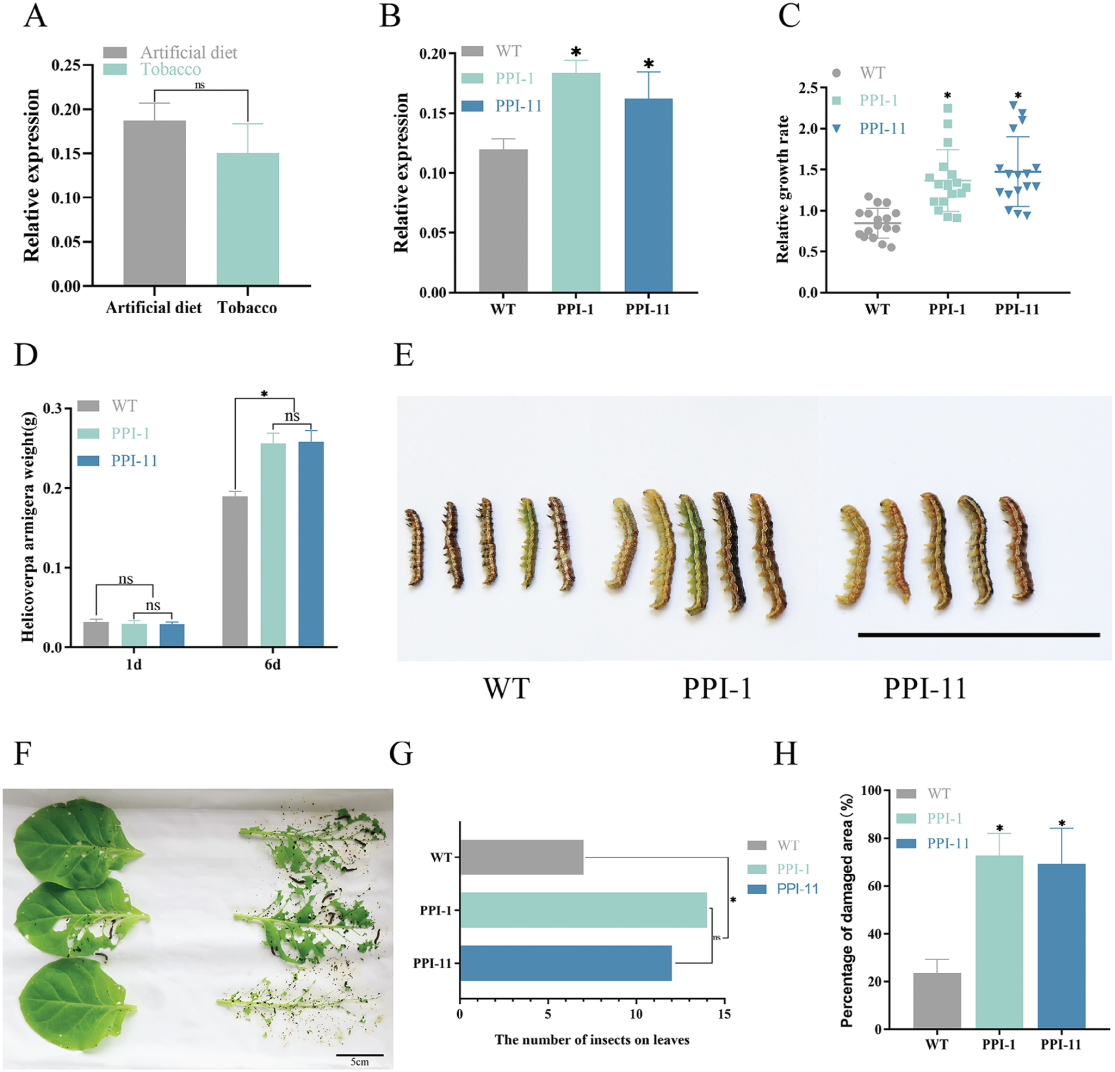
Overexpression of *PPI5* in tobacco‐promoted bollworm feeding. A) *PPI5* expression of cotton bollworm after feeding on artificial diet and WT tobacco. Artificial diet (AD): the diet of the cotton bollworm consists primarily of corn flour, wheat germ, soya flour, and a few vitamins. B) *PPI5* expression of cotton bollworm after feeding on WT tobacco, *PPI‐1*, and *PPI‐11*. C–E) The relative growth rate, body weight, and body size of cotton bollworm larvae fed on WT, *PPI‐1*, and *PPI‐11* tobacco. F–H) Preference feeding experiment of cotton bollworm. G) The number of cotton bollworms on WT, *PPI‐1*, and *PPI‐11* tobacco. H) Ratio of damage area to blade area after cotton bollworm larvae fed on *PPI‐1* and *PPI‐11*. Error bar, ±SD (*n  =*  3 biological replicates, 12–18 larvae were used as one replicate). Significance was determined by the Student's t‐test. *p < 0.05. Scale bars, 5 cm.

To further investigate the role of *PPI5* in insect and host plant interaction, 35S: PPI5 was heterologously expressed in *G. hirsutum* and *Nicotiana tabacum*. The independent transgenic lines of *G. hirsutum* (*PPI‐M and PPI‐N*) and *Nicotiana tabacum* (*PPI‐1* and *PPI‐11*) were selected for further study (Figure S5, Supporting Information). No significant difference was observed in *PPI5* transcription between larvae fed on an artificial diet (AD) and wild‐type (WT) plants of *N. tabacum* (**Figure** **3**A). However, the transcription of *PPI5* was significantly increased after feeding on two transgenic *N. tabacum* lines, *PPI‐1* and *PPI‐11*, as compared to the WT *N. tabacum* (Figure 3B). The relative growth rate, body weight, and body size of cotton bollworm larvae fed on *PPI‐1* and *PPI‐11* significantly increased compared to those fed on the WT *N. tabacum* (Figure 3C–E). We then performed a preferential feeding experiment in which 20 cotton bollworm larvae were randomly placed around WT, *PPI‐1*, and *PPI‐11* leaves to observe their feeding behavior. An average of 14 cotton bollworms preferred to feed on *PPI‐1* and *PPI‐11* overexpressed *N. tabacum* leaves compared to WT, while only 7 cotton bollworms on average fed on WT (Figure 3F,G). The results of the leaf area after feeding showed that the cotton bollworm fed on ≈23% of the WT *N. tabacum* area. Interestingly, the highest area of leaves fed on *PPI‐1* and *PPI‐11* ranged between 68 and 72% (Figure 3H; Figure S6, Supporting Information). These results confirmed that PPI5 acts as an effector to make *N. tabacum* more sensitive to cotton bollworm infestation.

**Figure 4:**
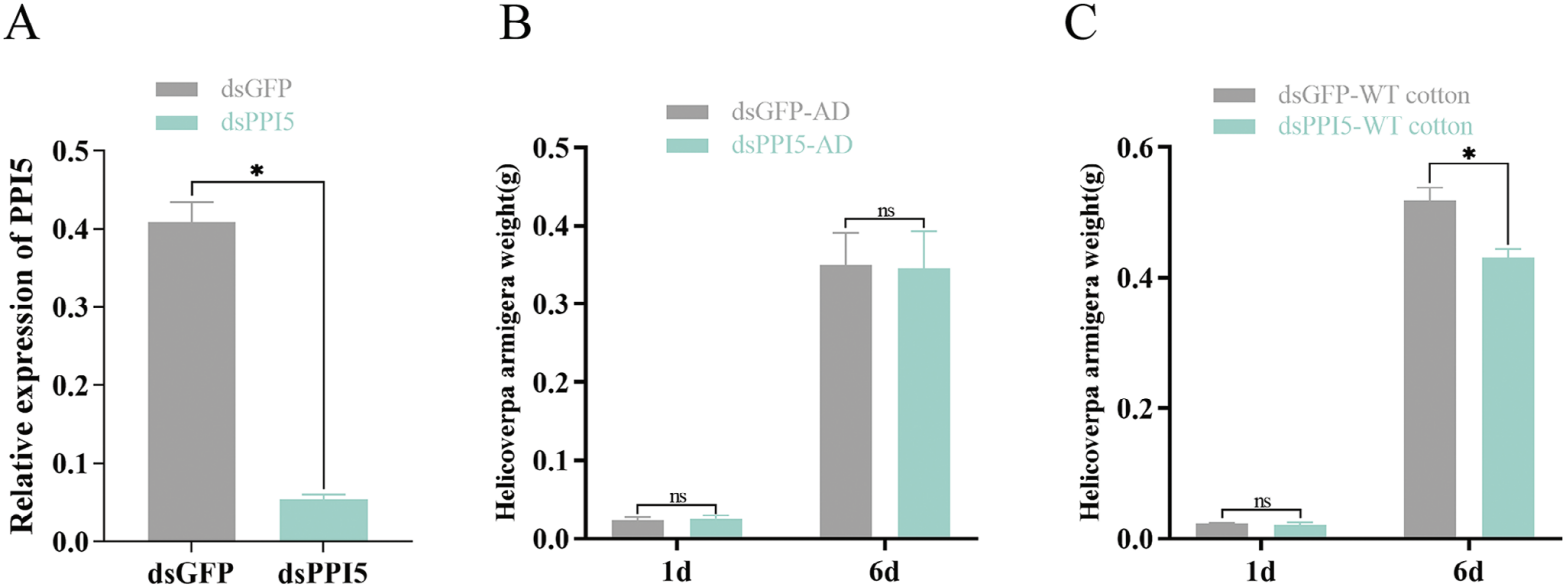
Silent *PPI5* expression in cotton bollworm affected its feeding on cotton. A) The relative expression of *PPI5* in the second instar larvae injected with dsPPI and dsGFP. B,C) Changes in body weight of cotton bollworm injected with dsGFP and dsPPI after feeding on AD and cotton leaves for 6d (day). Error bar, ±SD (*n  =*  3 biological replicates, 25–30 larvae were used as one replicate). Significance was determined by the Student's t‐test. *p < 0.05.

**Figure 5:**
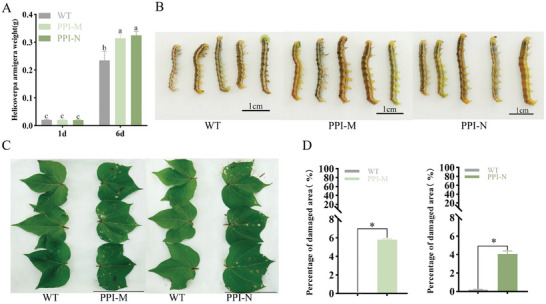
Overexpression of *PPI5* in cotton promoted bollworm feeding. A,B) Changes in body weight and body size of cotton bollworm after feeding on WT, *PPI‐M*, and *PPI‐N* cotton. Error bar, ±SD (*n  =*  3 biological replicates, 12–18 larvae were used as one replicate). Significance was determined by one‐way ANOVA. *p <* 0.05. C,D) Preference feeding experiment of cotton bollworm. The ratio of the damaged area to blade area after cotton bollworm larvae fed on WT, *PPI‐M*, and *PPI‐N* cotton. Images of cotton bollworm fed on WT, *PPI‐M*, and *PPI‐N* cotton 24 h. Error bar, ±SD (*n  =*  3 biological replicates, 10–18 larvae were used as one replicate). Significance was determined by the Student's t‐test. *p < 0.05. Scale bars, 5 cm.

We injected dsRNA into second instar larvae to interfere with the transcription of *PPI5* in cotton bollworm, to investigate whether *PPI5* affects the growth and development of cotton bollworm. Compared to dsGFP (control group), the transcription of *PPI5* decreased significantly after injecting dsPPI5 into cotton bollworms (**Figure** **4**A). Cotton bollworms injected with dsGFP and dsPPI5 did not differ significantly in their feeding behavior on AD (Figure 4B). However, cotton bollworms with dsPPI5 fed on WT cotton leaves showed a significantly lower weight than those of dsGFP (Figure 4C). These data further support the view that *PPI5* plays a key role in cotton bollworm invasion of cotton. To elucidate the role of *PPI5* in the host plant cotton, bioassay experiments were performed using WT and transgenic cotton (*PPI‐N*). Cotton bollworms fed on *PPI‐M* and *PPI‐N* cotton leaves were significantly heavier and larger than those fed on WT cotton leaves (**Figure** **5**A,B). The preferential feeding of the cotton bollworm on cotton leaves was measured and the results showed that the bollworms hardly fed on the WT cotton. Interestingly, the cotton bollworm caused a leaf area loss of 4–5.8% in *PPI‐1* and *PPI‐11* cotton, with significant differences noted compared to the damage caused by the same species feeding on WT cotton (Figure 5C,D; Figure S7, Supporting Information). These results suggest the expression of *PPI5* in tobacco and cotton increases the susceptibility of tobacco and cotton to cotton bollworm.

### 2.4 *PPI5* Inhibits the Defense Response of Cotton and Tobacco

**Figure 6:**
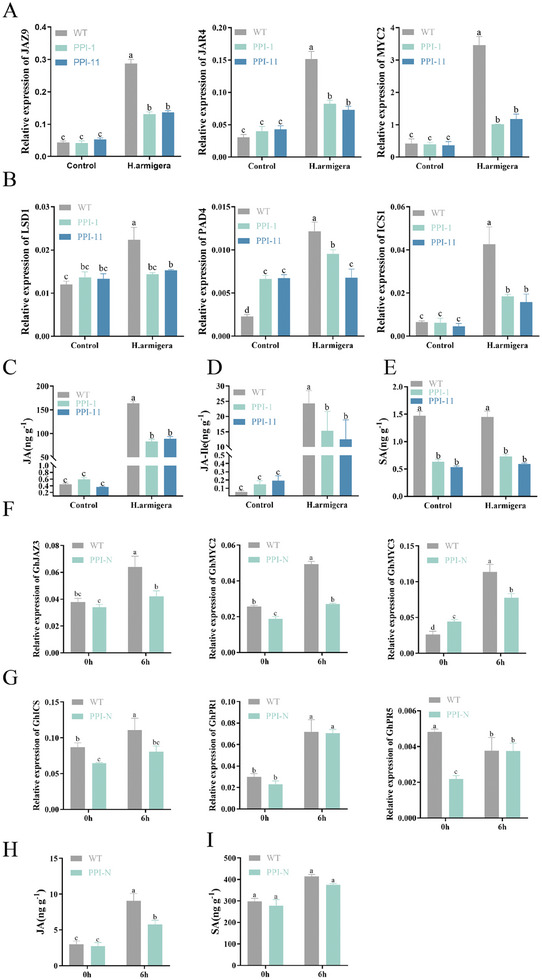
SA and JA levels and transcriptional levels of SA and JA related genes in cotton and tobacco after feeding induction of cotton bollworm. A,F) Expression levels of JA‐related genes in cotton and tobacco. B,G) Expression levels of SA‐related genes in cotton and tobacco. C–E) JA, JA‐Ile, and SA levels in tobacco. H,I) JA, and SA levels in cotton. Error bar, ±SD/SEM (*n  =*  3–4 biological replicates). Significance was determined by one‐way ANOVA. *p <* 0.05.

Insect feeding or mechanical damage triggers a set of plant stress responses, including the activation of different hormonal signaling pathways. To further investigate how *PPI5* increases susceptibility in cotton and tobacco, we therefore allowed cotton bollworms to feed freely on leaves of *PPI‐1*, *PPI‐11*, and *PPI‐N*, and JA and SA concentrations, and the expression levels of their related genes, were determined. Compared to WT, the expression of JA‐related genes *GhJAZ3* and *GhMYC2* was lower in *PPI‐N*, but for *JAZ9, JAR4*, and *MYC2* there was no significant difference found between *PPI‐1* and *PPI‐11* (**Figure** **6**A,F). JA‐related genes were significantly up‐regulated in tobacco and cotton after 6 h of cotton bollworm feeding, but interestingly, the JA‐related genes *JAZ9, JAR4, MYC2, GhJAZ3, GhMYC2* and *GhMYC3* were less induced in transgenic tobacco (*PPI‐1* and *PPI‐11*) and cotton (*PPI‐N*) plants after cotton bollworm feeding than that in wild‐type plants (Figure 6A,F). JA and JA‐Ile levels in transgenic tobacco and cotton are consistent with the expression of JA‐related genes (Figure 6C,D,H). Compared to WT cotton plants, expression of SA‐related genes *GhICS* and *GhPR5* was significantly suppressed in *PPI‐N* and there was no significant difference in *GhICS* and *GhPR5* expression after feeding by cotton bollworms (Figure 6G). SA levels in WT and *PPI‐N* were not significantly different (Figure 6I). Surprisingly, the accumulation of SA and expression of SA‐related genes (*LSD1*, *ICS1*) was not significantly altered by *PPI‐1* and *PPI‐11*, whereas SA levels and SA‐related gene expression (*LSD1*, *PAD4*, *ICS1*) were significantly down‐regulated in *PPI‐1* and *PPI‐11* compared to the WT after cotton bollworm feeding (Figure 6B,E). These results further confirmed that *PPI5* acts as an effector to attenuate plant defense to cotton bollworm. In addition, *PPI5* may regulate defense responses by acting in different ways in cotton and tobacco.

### 2.5 PPI5 Directly Interacts With the Cotton Cyclophilin GhFKBP17‐2

**Figure 7:**
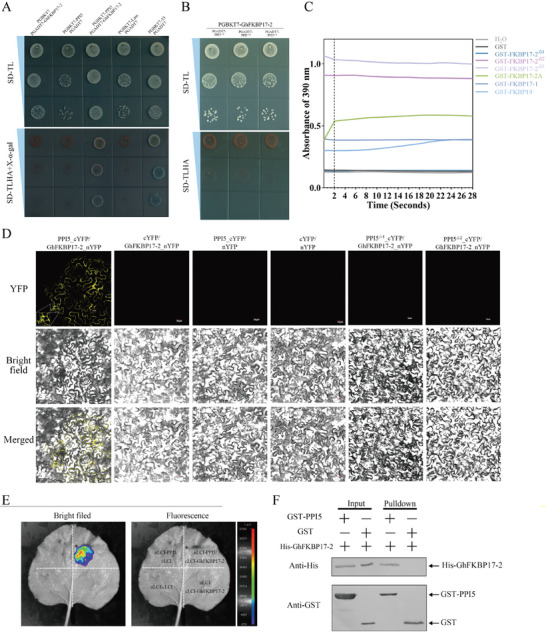
PPI5 directly interacts with GhFKBP17‐2. A) Yeast two‐hybrid assay. PPI5 was fused to the GAL4 DNA‐binding domain, to form PGBKT7‐PPI5. GhFKBP17‐2 was fused to the GAL4 activation domain, to form PGADT7‐ GhFKBP17‐2. Interactions were examined in SD‐TLHA (SD‐Leu‐Trp‐His‐Ade) +X‐α‐gal medium. SD‐TL: SD‐Leu‐Trp. B) Y2H assays to detect the interaction between GhFKBP17‐2 and PPI5 mutants (PPI5^△1^, PPI5^△2^, PPI5^△3^). C) Y2H assays to detect the interaction between PPI5 and GhFKBP17‐2 mutants (GhFKBP17‐2^△1^, GhFKBP17‐2^△2^, GhFKBP17‐2^△3^). D) Schematic diagram showing the protein structures of GhFKBP17‐2 and its deletion mutants (GhFKBP17‐2^△1^, GhFKBP17‐2^△2^, GhFKBP17‐2^△3^). Structural domains of GhFKBP17‐2 as analyzed by the NCBI and UniProt. The green part is the structural domain of GhFKBP17‐2. Numbers underneath each construct indicate amino acid positions. E) PPIase activity of prokaryotic expression of purified protein from GhFKBP17‐2 mutants and GhFKBP homologous genes. PPIase activities were analyzed by a chymotrypsin‐coupled assay using succinyl‐Ala‐Leu‐Pro‐Phe‐paranitroanilide as the substrate. The faster appearance of the absorbance at 390 nm is indicative of higher PPIase activity. GST and H_2_O, which served as a negative control. *Ghir_A08G018190.1*: *GhFKBP17‐2A*; *Ghir_D05G014750.1*: *GhFKBP17‐1*; *Ghir_D08G006560.1*: *GhFKBP20*; *Ghir_D04G015200.1*: *GhFKBP19*. F) Bimolecular fluorescence complementation (BiFC) assay verifying the interaction of GhFKBP17‐2, PPI5, PPI5^△1^, and PPI5^△2^. White dashed boxes indicate YFP fluorescence was observed from ER. Scale bars, 30 µm. G, luciferase complementation imaging (LCI) assay of cLCI‐PPI5 and nLCI‐GhFKBP17‐2. cLCI and cLCI‐PPI5 were co‐injected with nLCI and nLCI‐GhFKBP17‐2, respectively, and luciferin signals were captured using a whole‐body fluorescent imaging system. H, In pull‐down assay, GST‐PPI5, but not GST, is co‐immunoprecipitated with His‐GhFKBP17‐2. Histidine (His)‐ GhFKBP17‐2, glutathione S‐transferase (GST)‐PPI5, and GST were prokaryotically expressed and purified. His‐GhFKBP17‐2 was incubated with GST and GST‐PPI5. The amount of His‐GhFKBP17‐2 in input was visualized and quantified by WB. Anti‐His and anti‐GST antibodies were used to detect His and GST‐fused proteins, respectively, in putdown.

To investigate how *PPI5* regulates hormone levels in cotton and tobacco, we used PPI5 to screen yeast activation domain libraries of cotton infested by *H. armigera*, *Spodoptera litura*, and whitefly. Ten cotton endogenous genes were screened from the libraries (Figure S8A,B, Supporting Information). After further yeast two‐hybrid point‐to‐point validation, we found that *Ghir_D08G019080.1* (GhFKBP17‐2) and PPI5 showed positive interaction (**Figure** **7**A). In addition, we verified the reciprocal relationship between *PPI5* mutants (PPI5^△1^; PPI5^△2^; PPI5^△3^) and the *GhFKBP17‐2* mutants (pGADT7‐GhFKBP17‐2^△2^/ pGADT7‐GhFKBP17‐2^△3^). As the yeast strain containing bait (pGBDT7‐GhFKBP17‐2) and prey (pGADT7‐PPI5^△1^/pGADT7‐PPI5^△2^/ pGADT7‐PPI5^△3^) failed to grow in SD‐TLHA (Figure 7B). Conversely, yeast strains containing the bait (pGBDT7‐PPI5) and prey (pGADT7‐GhFKBP17‐2^△2^/ pGADT7‐GhFKBP17‐2^△3^) grew successfully in SD‐TLHA (Figure 7C). We have characterized that both GhFKBP17‐2^△2^ and GhFKBP17‐2^△3^ contain the structural domain of PPIase (Figure 7D).

FKBPs belong to the cyclophilins, which possess PPIase activity essential for its contribution to immunity.^[^ ^43^, ^77^ ^]^ We then confirmed that GhFKBP17‐2 possesses PPIase activity by the chymotrypsin‐coupled assay using succinyl‐Ala‐Leu‐Pro‐Phe‐paranitroanilide as the alpha‐chymotrypsin substrate.^[^ ^43^, ^44^ ^]^ *GhFKBP17‐2A*, *GhFKBP17‐1* and *GhFKBP19* are homologues genes of *GhFKB17‐2*. The GST fusion proteins, GST, GST‐GhFKBP17‐2^△1^, GST‐GhFKBP17‐2^△2^, GST‐GhFKBP17‐2^△3^, GST‐GhFKBP17‐2A, GST‐GhFKBP17‐1 and GST‐GhFKBP19, were expressed in *Escherichia coli* and purified by binding to GST‐Sefinose resin columns, and protein expression was confirmed by both SDS–PAGE (Figure 10A). Compared to GST, GST‐GhFKBP17‐2^△2^ and GST‐GhFKBP17‐2^△3^ protein purified from *E. coli* showed clear PPIase activity, GST‐GhFKBP17‐2^△1^ purified protein lost the PPIase activity (Figure 7E). These results indicate that the structural domain of *GhFKBP17‐2* plays a key role in mediating the interaction between GhFKBP17‐2 and PPI5. The amino acid identity of the *FKBP* family ranged from 10% (lowest) to 68.4% (highest) in the *Arabidopsis*.^[^ ^83^ ^]^ To test whether the *GhFKBP* functional domain is specific and conserved. We performed an evolutionary tree construction of the FKBP family in cotton, and the results showed that *Ghir_A08G018190.1* (*GhFKBP17‐2A*) was homologous to *GhFKBP17‐2* (Figure 9B). In the chymotrypsin‐coupled assay, GST‐GhFKBP17‐2A, GST‐GhFKBP17‐1, and GST‐GhFKBP19 exhibited PPIase activity when compared to the spontaneous reaction with water (H_2_O). However, their PPIase activity was considerably lower than that of GST‐GhFKBP17‐2 functional mutants (Figure 7E). These results demonstrate that GhFKBP17‐2 is a canonical cyclophilin with the PPIase activity.

To test whether the interactions between *PPI5* and *GhFKBP17‐2* are specific and conserved. We used PPI5 as a bait protein to interact with GhFKBP17‐2A, GhFKBP17‐1, GhFKBP20, and GhFKBP19 for yeast point‐to‐point validation. We found that PPI5 exhibited significant binding activity only to GhFKBP17‐2A (Figure 9C,D). With 97.9% homology, GhFKBP17‐2A differs from GhFKBP17‐2 by only 5 amino acids (Figure S9A, Supporting Information). We found that both PPI5 and GhFKBP17‐2 are proline cis‐trans isomerases, and we speculated that PPI5 and GhFKBP17‐2 would interact with themselves. The PPI5, *GhFKBP17‐2*, and *GhFKBP17‐2A* were fused with the activation domain (pGADT7) and the binding domain (pGBKT7). The yeast two‐hybrid (Y2H) assay results showed that PPI5 and GhFKBP17‐2 did not interact with each other by themselves, and GhFKBP17‐2 and GhFKBP17‐2A did not interact with each other (Figure 9E–G).

Subsequently, the interaction was validated using luciferase complementation imaging (LCI) assay and bimolecular fluorescence complementation (BiFC) assay in *N. benthamiana* leaves. In the BiFC assay, a yellow fluorescence signal of positive interactions was observed between PPI5 and GhFKBP17‐2 (Figure 7F). However, co‐transfection between the empty vector (cYFP) with GhFKBP17‐2‐nYFP; the empty vector (nYFP) with PPI5‐nYFP; cYFP with nYFP and the fusion vector (PPI5^△1^‐cYFP, PPI5^△2^‐cYFP) with GhFKBP17‐2‐nYFP did not produce positive signals (Figure 7F). The coding sequence of *PPI5* and *GhFKBP17‐2* were linked to the N‐ and C‐termini of luciferase respectively for analysis by Luciferase Complementation Imaging (LCI) following co‐transformation in *N. benthamiana* leaves. After two days of co‐infection, nLCI‐PPI5, and cLCI‐GhFKBP17‐2 resulted in fluorescence, while other combinations could not (Figure 7G). We further constructed prokaryotic expression vectors pGEX‐4T‐1 and PET‐28‐a with *PPI5* and *GhFKBP17‐2* to induce GST‐PPI5 and His‐GhFKBP17‐2 proteins. The purified GST‐PPI5 and His‐GhFKBP17‐2 proteins were used for pull‐down assay (Figure S11, Supporting Information) and results showed that GST‐PPI5 could be co‐precipitated with His‐FKBP17‐2, but GST could not be co‐precipitated with His‐FKBP17‐2 (Figure 7H). These results indicate a direct interaction between PPI5 and GhFKBP17‐2.

### 2.6 PPI5 and GhFKBP17‐2 co‐localize in the ER

**Figure 8:**
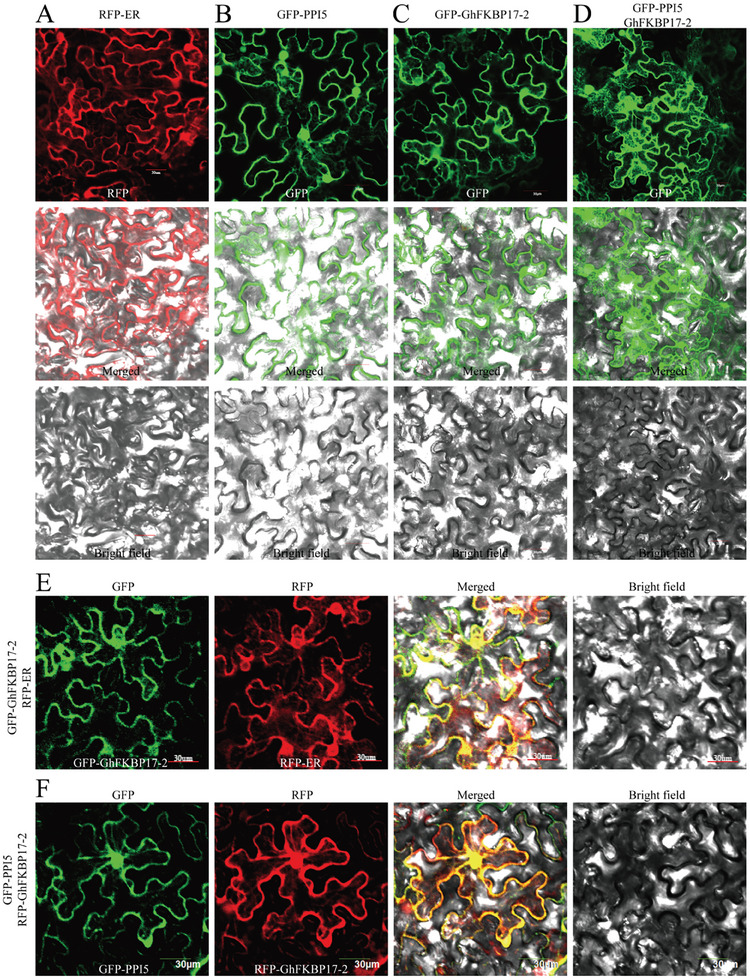
PPI5 and GhFKBP17‐2 co‐localize to the ER. In all panels, proteins were expressed in *N. benthamiana* through Agroinfiltration. Fluorescence in *N. benthamiana* epidermal cells was observed by confocal microscopy at 48 h post infiltration. Scale bars, 30 µm. A) RFP‐ER, B) GFP‐PPI5, and C) GFP‐ GhFKBP17‐2 subcellular localization in the ER. D) When MYC‐GhFKBP17‐2 and GFP‐PPI5 were co‐expressed, PPI5 fluorescence was stronger on the ER. E) GFP‐GhFKBP17‐2 co‐localizes with RFP‐ER. F, GFP‐GhFKBP17‐2, and RFP‐PPI5 co‐localize to the ER.

To investigate the subcellular localization of *GhFKBP17‐2* and its association with PPI5, the localization patterns of the proteins were determined in plant cells. We constructed plasmids to express fusion proteins: 35S: GFP/RFP‐PPI5 (PPI5 SP was removed) and 35S: MYC/GFP‐GhFKBP17‐2. RFP fluorescence in RFP‐labeled ER (RFP‐ER) marker expressing *N. benthamiana* localized with ER‐like networks and surrounded the nucleus (**Figure** **8**A). We found that GFP‐PPI5 localized in an ER‐like network of subcellular structures including the perinuclear ER (Figure 8B). Consistent with the previously observed localization of FKBP to the ER,^[^ ^43^ ^]^ GFP‐GhFKBP17‐2 were detected to be localized in an ER‐like network of subcellular structures including the perinuclear ER (Figure 8C). Co‐expression of GFP‐GhFKBP17‐2 with an ER marker (RFP‐ER) confirmed that the localization was to the ER (Figure 8E). Notably, when GFP‐PPI5 was co‐expressed with MYC‐GhFKBP17‐2 in *N. benthamiana*, GFP‐PPI5 was localized in the ER network including the perinuclear ER (Figure 8D). Moreover, the infiltrated leaves expressing GFP‐PPI5 co‐expressed with MYC‐GhFKBP17‐2 showed stronger fluorescence in contrast to those expressing GFP‐GhFKBP17‐2 or GFP‐PPI5 (Figure 8B–D). Surprisingly, GFP‐GhFKBP17‐2 completely overlapped with the RFP‐PPI5 in the peri‐nuclear ER and the ER network when these proteins were co‐expressed in *N. benthamiana* leaves (Figure 8F). Taken together, these results suggest that PPI5 can be co‐localized with *GhFKBP17‐2* in the ER, and the PPI5 and GhFKBP17‐2 interaction occurs in the ER.

### 2.7 Different Modes of Action of PPI5 in Regulating the *FKBP* Response in Cotton and Tobacco

Overexpression of *PPI5* in cotton and tobacco promotes cotton bollworm feeding and downregulates plant JA levels and JA‐related gene expression (Figure 6). Further experiments revealed that PPI5 interacts with cotton GhFKBP17‐2, but how PPI5 regulates *GhFKBP17‐2* remains unclear (Figure 7). Therefore, the expression of *FKBP* was examined in *PPI‐1*, *PPI −11* and *PPI‐N* transgenic plants. There was a significant difference in the transcription level of *FKBP* in *PPI‐1* and *PPI‐11* under normal conditions, but after cotton bollworm feeding, the transcription of *FKBP* in *PPI‐1* and *PPI‐11* was significantly decreased compared to the WT tobacco plants (Figure S12A, Supporting Information). *GhFKBP17‐2* expression was significantly suppressed in *PPI‐N* compared to WT cotton under normal conditions; however, the transcript level of *GhFKBP17‐2* was significantly upregulated when feeding was induced by cotton bollworms (Figure S12B, Supporting Information).

### 2.8 PPI5 Directly Suppresses the PPIase Activity of GhFKBP17‐2

**Figure 9:**
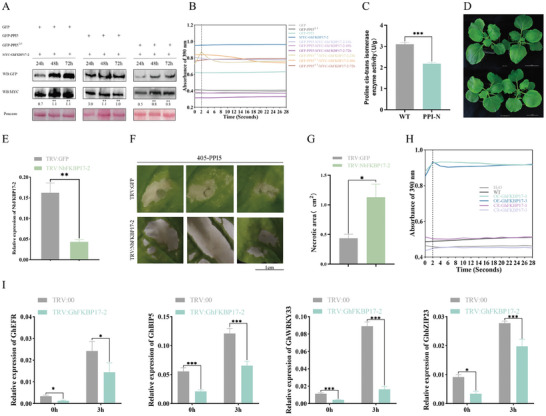
The PPIase activity of GhFKBP17‐2 is required for its immune function during PPI5 infection. A) Protein stability of GhFKBP17‐2, co‐expressed with GFP, GFP‐PPI5, or GFP‐PPI5^Δ1^, was analyzed by immunoblotting (IB). The MYC‐GhFKBP17‐2 protein was co‐expressed with GFP, GFP‐PPI5, or GFP‐PPI5^Δ1^, in *N. benthamiana* leaves through agroinfiltration. Total proteins were extracted from infiltrated leaves at 24, 48, and 72 h post agroinfiltration. MYC‐GhFKBP17‐2, GFP, GFP‐PPI5, or GFP‐PPI5^Δ1^ were detected by immunoblotting using anti‐MYC‐ and GFP‐antibodies, respectively. Ponceau staining of the membrane was used to show equal loading. B) PPIase activity of MYC‐GhFKBP17‐2 protein co‐expressed with GFP, GFP‐PPI5, or GFP‐PPI5^Δ1^. PPIase activities were analyzed by chymotrypsin‐coupled assay at 8 °C, using succinyl‐Ala‐Leu‐Pro‐Phe‐paranitroanilide as the substrate. The faster appearance of the absorbance at 390 nm is indicative of higher PPIase activity. C) Determination of peptidylproline cis‐trans isomerase (PPI) enzyme activity in transgenic material by ELISA kit. Read the Optical Density (O.D.) at 450 nm using a microtiter plate reader within 15 min. D) Silencing of *N. benthamiana FKBP17‐2* by VIGS. VIGS assays were conducted at the 4‐leaf stage of *N. benthamiana*. Symptoms of *N. benthamiana* after silencing were photographed after 5 weeks of growth. Scale bars, 5 cm. E) Silencing efficiency of NbFKBP17‐2 in the indicated gene knock‐down *N. benthamiana* plants (*n =* 3). F) Transient expressing PPI5 in *GFP*‐ and *NbFKBP17‐2* ‐silenced *N. benthamiana* leaves. Inhibition of *NbFKBP17‐2* expression in *N. benthamiana* enhanced the infection by PPI5. Necrotic area statistics as shown by images (F) and G) statistical analysis at 72 h after agroinfiltration. Error bars represent SEM. H) PPIase activity of transgenic cotton. PPIase activities were analyzed by chymotrypsin‐coupled assay. I) Expression levels of *GhEFR*, *GhbZIP23*, *GhWRKY33*, and *GhBiP5* were determined by qRT–PCR. Detached leaves of 4‐week‐old plants of *TRV:00* and *TRV: GhFKBP17‐2* plants were inoculated with GST‐PPI5. Total RNA was extracted from leaves at 0 and 3 h. Error bar, ±SD. Asterisks represent statistically significant differences from Student's t‐tests (*p < 0.05, **p < 0.01, ***p < 0.001).

Previous reports revealed that the *Phytophthora capsici* effector PcAvr3a12, the *Bursaphelenchus xylophilus* effector BxML1, and the *Xanthomonas citri* effector PthA2 facilitate infection and virulence by targeting and suppressing the PPIase activity of CYP.^[^ ^43^, ^76^, ^78^ ^]^ We next investigated whether the abundance of *GhFKBP17‐2* could be affected by PPI5. For this purpose, we compared the accumulation of MYC‐GhFKBP17‐2 in *N. benthamiana* when co‐expressed with GFP, GFP‐PPI5, or GFP‐PPI5^Δ1^ using *Agroinfiltration*. We found that the accumulation of MYC‐GhFKBP17‐2 was significantly decreased in the leaves co‐expressing GFP‐PPI5, and MYC‐GhFKBP17‐2 accumulated to a much higher level in the presence of GFP or GFP‐PPI5^Δ1^ (**Figure** **9**A). PPIase activity was further detected. PPIase activities were analyzed using chymotrypsin‐coupled assays. A higher absorbance at 390 nm indicates increased PPIase activity. Compared to GFP, the addition of GFP‐PPI5 resulted in a faster accumulation of 4‐nitroaniline and a higher absorbance at 390 nm; the absorbance at 390 nm of GFP‐PPI5^Δ1^ was consistent with that of GFP, suggesting that GFP‐PPI5 has PPIase activity (Figure 9B). The PPIase activity of MYC‐GhFKBP17‐2 was observed to be higher in *N. benthamiana* when MYC‐GhFKBP17‐2 was co‐expressed with GFP‐PPI5^Δ1^ for 24–72 h, or with GFP‐PPI5 for 24 h (Figure 9B). It is noteworthy that the PPIase activity of MYC‐GhFKBP17‐2 was found to be diminished when MYC‐GhFKBP17‐2 was co‐expressed with GFP‐PPI5 for 48 to 72 h in *N. benthamiana* (Figure 9B).

PPIase activity was further detected in WT and *PPI‐N* transgenic plants. Compared with the WT, the PPIase activity of *PPI‐N* was significantly decreased (Figure 9C). These results provide evidence that the presence of PPI5 can reduce the accumulation and PPIase activity of GhFKBP17‐2.

### 2.9 Knockdown of *NbFKBP17‐2* Enhances PPI5‐induced Cell Death

To determine plant regulators associated with PPI5‐mediated cell death, we knocked down the expression of *NbFKBP17‐2* with a VIGS assay in *N. benthamiana* (Figure 9D,E). qRT–PCR analysis revealed that the expression of *NbFKBP17‐2* was considerably reduced in silenced plants compared with control plants (Figure 9E). When infected with PPI5, the sizes of necrotic areas on *NbFKBP17‐2*‐silenced leaves were significantly bigger than those on the non‐silenced leaves (Figure 9F,G).

### 2.10 *GhFKBP17‐2* is Involved in ER Stress‐Mediated Plant Immunity

CYPs *AtFKBP65*, *MdFKBP62a*, *MdFKBP65a/b*, *ROC3*, *ROF2*, *AtCYP57*, and *PaFKBP12* confer stress tolerance to biotic and abiotic.^[^ ^71^, ^72^, ^84^, ^85^ ^]^ We hypothesized that cotton and tobacco activate the JA and SA pathways by up‐regulating *FKBP* expression, while cotton bollworm *PPI5* inhibits the downstream defense response by suppressing *FKBP* transcription. In order to explore how *PPI5* regulates *GhFKBP17‐2*, *GhFKBP17‐2* overexpression (35S: GhFKBP17‐2) and knockout (Cas9: GhFKBP17‐2) vectors were constructed for *Agrobacterium*‐mediated genetic transformation in cotton (Figure S13A–D,G,I, Supporting Information). Immunoblotting analyses revealed that three OE transgenic lines expressed the correct molecular weight of the GhFKBP17‐2 protein (Figure S13F, Supporting Information). Two OE lines (*OE‐GhFKBP17‐1* and *OE‐GhFKBP17‐3*) with high expression levels were selected for detailed analysis (Figure S13E, Supporting Information). Furthermore, we created two mutant lines *CR‐GhFKBP17‐1* and *CR‐GhFKBP17‐3* using CRISPR/Cas9‐mediated gene editing. These were confirmed by high‐throughput sequencing as frame‐shift mutations and were selected for further insect bioassay (Figure S13H,I, Supporting Information).

Here, a conventional protease‐coupled PPIase assay was applied to determine whether *GhFKBP17‐2* has PPIase activity and whether it plays an important role in plant immunity. Total protein from transgenic cotton lines *OE‐GhFKBP17‐1/3* and *CR‐GhFKBP17‐1/3* was extracted and analyzed by SDS‐PAGE and immunoblotting (Figure S13F, Supporting Information). PPIase activities were analyzed by chymotrypsin‐coupled assays. Compared with the spontaneous reaction of H_2_O, the accumulation of 4‐nitroaniline was faster and absorbance at 390 nm was higher with the addition of *OE‐GhFKBP17‐1* or *OE‐GhFKBP17‐3*; the absorbance of 390 nm of *CR‐GhFKBP17‐1* and *CR‐GhFKBP17‐3* were consistent with the spontaneous reaction of H_2_O, indicating that *OE‐GhFKBP17‐1/3* possesses higher PPIase activity (Figure 9H).

Our findings that GhFKBP17‐2 is localized in the ER and has PPIase activity prompted us to question whether *GhFKBP17‐2* mediates immunity against insects by regulating ER stress. To investigate whether the contribution of *GhFKBP17‐2* to immunity is related to its contribution to ER stress and UPR regulation, we examined the transcript levels of the ER stress sensor genes *GhEFR*, *GhbZIP23*, *GhWRKY33*, and *GhBiP5* during *PPI5* infection. To test this, virus‐induced gene silencing (VIGS) was used to knock down the transcription of *GhFKBP17‐2* (Figure S15A,B, Supporting Information). A recombinant *pTRV2* vector, *TRV: GhFKBP17‐2*, was constructed and the empty recombinant *pTRV2* vector (*TRV:00*) was used as a control. In the *TRV: GhFKBP17‐2* plants, the transcription of *GhEFR, GhbZIP23, GhWRKY33*, and *GhBiP5* was significantly reduced compared with that in *TRV:00* plants (Figure 9I). When GST‐PPI5 was infected for 3 h, transcript levels of the ER stress sensor genes were significantly reduced compared with that in *TRV:00* (Figure 9I). Taken together, these results imply that *GhFKBP17‐2* contributes to ER stress‐mediated plant immunity.

### 2.11 The *GhFKBP17‐2* is Essential for Plant Resistance to Cotton Bollworm Invasion

**Figure 10:**
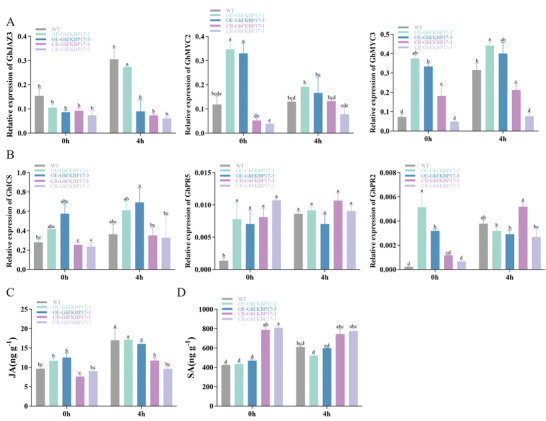
JA, SA content, JA‐, SA‐related gene expression of WT, OE‐GhFKBP17‐1/3 and CR‐GhFKBP17‐1/3 cotton. A) Transcriptional levels of JA‐related genes in WT and transgenic cotton (WT, OE‐GhFKBP17‐1/3 and CR‐GhFKBP17‐1/3). B) Transcriptional levels of SA‐related genes in cotton. C,D) JA, and SA levels in cotton. Error bar, ±SD/SEM (*n  =*  3–4 biological replicates). Significance was determined by one‐way ANOVA. *p <* 0.05.

To investigate the role of *GhFKBP17‐2* in plant defense, JA and SA levels and transcript levels of JA‐ and SA‐responsive genes were determined in WT, *OE‐GhFKBP17‐1/3* and *CR‐GhFKBP17‐1*/*3* plants. The expression levels of JA‐related genes (*GhMYC2, GhMYC3*) and SA‐related genes (*GhICS, GhPR2, GhPR5*) were significantly higher *in OE‐GhFKBP17‐1/3* than in WT without cotton bollworm feeding, whereas in *CR‐GhFKBP17‐1/3* the transcription levels of JA‐related genes (*GhJAZ3*, *GhMYC2, GhMYC3*) and SA‐related genes (*GhICS, GhPR5*) were similar to WT (**Figure** **10**A,B). The transcription of JA‐related genes was up‐regulated 4 h after induction of cotton bollworm feeding. Notably, the transcription of JA‐related genes was similar in *OE‐GhFKBP17‐1/3* compared to WT, whereas the transcription of JA‐related genes was significantly lower in *CR‐GhFKBP17‐1/3* (Figure 10A). Transcription of SA‐related genes induced by cotton bollworm feeding at 4 h was not significantly different from non‐induced genes (Figure 10B). The levels of JA are consistent with the expression of JA‐related genes in WT, *OE‐GhFKBP17‐1/3*, and *CR‐GhFKBP17‐1/3* (Figure 10C). SA levels in *CR‐GhFKBP17‐1/3* were significantly higher than in WT before and after infection by cotton bollworm (Figure 10D). Transcription of JA‐ and SA‐related genes in the *TRV: GhFKBP17‐2* was consistent with that of *CR‐GhFKBP17‐2* (Figure S15G,H, Supporting Information).

**Figure 11:**
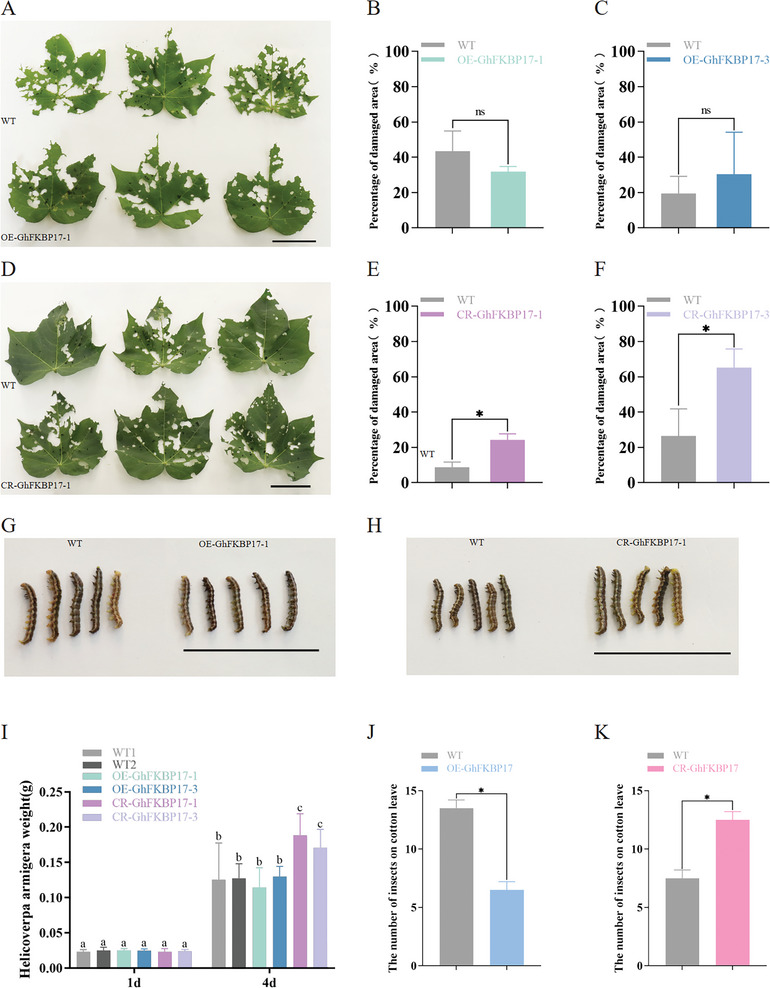
Bioassay of cotton bollworm on transgenic cotton in the greenhouse. A–F) Preference feeding experiment of cotton bollworm. B,C,E,F) Ratio of damage area to blade area after cotton bollworm larvae fed on WT cotton, OE‐GhFKBP17‐1/3, and CR‐GhFKBP17‐1/3. G–I) The body weight and body size of cotton bollworm larvae fed on WT cotton, OE‐GhFKBP17‐1, and CR‐GhFKBP17‐1. Significance was determined by one‐way ANOVA. *p <* 0.05. J,K) The number of cotton bollworms on WT, OE‐GhFKBP17, and CR‐GhFKBP17. Error bar, ±SD (*n  =*  3 biological replicates, 12–18 larvae were used as one replicate). Significance was determined by Student's t‐test and one‐way ANOVA. *p < 0.05. Scale bars, 5 cm.

We determined whether *OE‐GhFKBP17‐1/3* and *CR‐GhFKBP17‐1/3* transgenic cotton affected bollworm feeding behavior in greenhouses and in the field. Under greenhouse conditions, young cotton leaves of WT, *OE‐GhFKBP17‐1/3*, and WT, *CR‐GhFKBP17‐1/3* were placed evenly in square dishes padded with wet filter paper, and then 20 cotton bollworm larvae were placed evenly around the cotton leaves to allow the bollworm to feed randomly for 24 h. The number of bollworms distributed in different cotton leaves and the feeding area of the cotton leaves were counted (Figure S14, Supporting Information). Of 20 randomly fed bollworms, 13 were preferentially fed on WT, while only 7 preferred to feed on *OE‐GhFKBP17* (**Figure** **11**J). However, out of 20 larvae, ≈12 were more likely to feed *CR‐GhFKBP17*, while only 7 preferred to feed on WT leaves (Figure 11K). There was no significant difference in leaf area between *OE‐GhFKBP17‐1* and *OE‐GhFKBP17‐3* fed by cotton bollworm compared to WT (Figure 11A–C; Figure S14A–D, Supporting Information). Notably, the leaf damage area of *CR‐GhFKBP17‐1* and *CR‐GhFKBP17‐3* consumed by cotton bollworms was significantly increased by 15.51%–38.8% than the WT plants (Figure 11D–F; Figure S14E–J, Supporting Information). Accordingly, the body shape and body weight of cotton bollworms fed on different cotton materials were measured. There was no significant difference between the body weight and shape of cotton bollworms fed on *OE‐GhFKBP17‐1/3* and WT, whereas the body weight and shape when fed on *CR‐GhFKBP17‐1/3* was significantly greater than for WT (Figure 11G–I).

**Figure 12:**
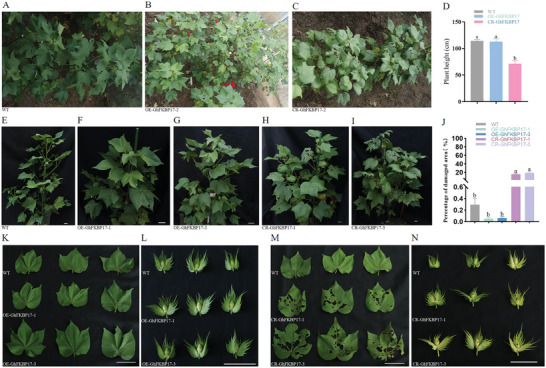
Bioassay of cotton bollworm on transgenic cotton in the field. A–C) In experimental plots, the growth and insect infestation of WT, *OE‐GhFKBP17‐2*, and *CR‐GhFKBP17‐2* T0 transgenic cotton. D) Plant height of WT, *OE‐GhFKBP17*, and *CR‐GhFKBP17* cotton. E–I) Individual plant growth and insect infestation of WT, *OE‐GhFKBP17‐1/3*, and *CR‐GhFKBP17‐1/3* cotton. J,K,M) The ratio of damage area to blade area of WT, *OE‐GhFKBP17‐1/3*, and *CR‐GhFKBP17‐1/3* cotton. L,N) Flower buds eaten by cotton bollworms of WT, *OE‐GhFKBP17‐1/3*, and *CR‐GhFKBP17‐1/3* cotton. Significance was determined by one‐way ANOVA. *p <* 0.05. Scale bars, 5 cm.

To evaluate the resistance of the transgenic cotton plants (*OE‐GhFKBP17‐1/3* and *CR‐GhFKBP17‐1/3*) to cotton bollworms under field conditions, transgenic plants were selected for field insect bioassays. Transgenic and control plants (WT) were planted in experimental plots that received no treatment other than fertilizer. Insects were allowed to develop naturally in the fields, and pests were counted. *H. armigera* prefers to feed on young tissues, such as tender leaves, shoot tips, and flowers, causing stunted plant growth and tissue abscission. Moreover, cotton bolls are also frequently attacked by *H. armigera*, resulting in boll damage and internal necrosis, all of which can cause severe yield loss. Therefore, plant height, leaf damage lesions, and flower abscission were recorded as typical phenotypes to quantify the *H. armigera* damage. Compared to the control plants, *OE‐GhFKBP17‐1/3* showed less insect damage, however, *CR‐GhFKBP17‐1/3* showed more traces of insect damage (**Figure** **12**A–C); *OE‐GhFKBP17* showed normal plant height, whereas the *CR‐GhFKBP17* showed a significant reduction in plant height by 37.9% (Figure 11D–J); the ratio of damage area to blade area of *CR‐GhFKBP17‐1/3* significantly decreased by 15.0%–18.5% compared to the control and *OE‐GhFKBP17‐1/3* plants (Figure 12J,K,M). In addition, flower buds of *CR‐GhFKBP17‐1/3* exhibited more pronounced cotton bollworm holes and were more likely to abort compared to control and *OE‐GhFKBP17‐1/3* plants (Figure 12L,N).

To provide further evidence that the cotton bollworm effector PPI5 attenuates the plant defense through *GhFKBP17‐2*, *TRV:00*, and *TRV: GhFKBP17‐2* plants were used to apply exogenous GST and GST‐PPI5 recombinant purified proteins. The expression of three protease inhibitors (*Gh_D06G1912*, *Gh_Sca005135G01*, and *Gh_D12G2247*), which are defense compounds induced by insect herbivory and mechanical injury, was investigated. The results showed that exogenous application of GST‐PPI5 protein to the leaves of cotton plants resulted in a clear attenuation of wounding response (Figure S13B, Supporting Information). By contrast, no obvious difference in the induction of proteinase inhibitor genes (*Gh_Sca005135G01* and *Gh_D12G2247*) by exogenous application of PPI5 between the *TRV: 00* and *TRV: GhFKBP17‐2* groups (Figure S13B, Supporting Information). In addition, the VIGS plants were used for cotton bollworm bioassays. The feeding behavior of cotton bollworms in the *TRV: GhFKBP17‐2* was consistent with that of *CR‐GhFKBP17‐2* (Figure S15C–F, Supporting Information). To further investigate specifically PPI5‐GhFKBP17‐2 interaction on cotton bollworm growth and development, dsGFP and dsPPI5 cotton bollworm (Figure 4A) feeding bioassays were observed in *TRV: 00* and *TRV: GhFKBP17‐2* plants. The weight and body size of dsPPI5 cotton bollworms were significantly reduced in *TRV: 00* plants (Figure S16C,D, Supporting Information). By contrast, there was no significant difference in larval growth between the dsGFP and dsPPI5 groups feeding on *TRV: GhFKBP17‐2* (Figure S16C,D, Supporting Information). The defense genes in cotton leaves had a higher induction level with the feeding of dsPPI5 bollworm than that of dsGFP bollworm feeding (Figure S16A, Supporting Information). Together, these data reveal that PPI5 inhibits the ER‐ and JA‐mediated immune response by suppressing the transcription and PPIase activity of *GhFKBP17‐2* in cotton, thus ensuring cotton bollworm growth and development.

## 3 Discussion

The *H. armigera* is a polyphagous generalist pest species that occurs worldwide. The cotton bollworm has invaded numerous countries across Asia, Africa, and Europe, largely due to its generalist nature, migratory adults, and high fecundity. This species of insect is capable of feeding on over 100 different plant species, resulting in significant economic losses to crops, with an estimated value exceeding $2 billion.^[^ ^86^ ^]^ The generalist herbivore *H. armigera* exhibits a plastic dietary pattern, feeding on a wide range of crops, including cotton, tobacco, and eggplant. In contrast, the closely related species *H. assulta* is a specialist herbivore with a narrower dietary preference, favoring tobacco.^[^ ^87^, ^88^ ^]^ Plant‐insect interaction studies underscore the adaptability and plasticity of insect‐feeding behaviors. For instance, *GOX* has been observed to regulate plant defense responses in tobacco and tomato in different manners.^[^ ^34^, ^88^, ^89^, ^90^ ^]^ The use of both cotton and tobacco in our study allows for a comprehensive comparison between a model plant and an important agricultural crop, enabling us to investigate the diverse functional roles of *PPI5* in different ecological and physiological contexts.

Over millions of years of insect‐plant co‐evolution, such insects have evolved long, thin mouthparts to suck plant sap to feed, causing only tiny wounds that induce plants to sense and respond to stress and secrete salivary effectors to weaken the plant defenses. However, chewing mouthpart insects cause significant mechanical damage, so the plant reacts quickly and strongly to prevent insect invasion. The insects, in turn, secrete various types of effectors that interfere with plant immunity through a range of regulatory mechanisms to survive and reproduce.^[^ ^4^, ^38^, ^39^ ^]^ Effectors RP191 and RP246 from *Riptortus pedestris* trigger the *N. benthamiana* hypersensitivity response but also induce plant sensitivity.^[^ ^91^ ^]^ *Apolygus lucorum* CYP Al106 inhibits PAMP‐induced cell death and ROS burst and promotes insect feeding and plant pathogen infection.

**Figure 13:**
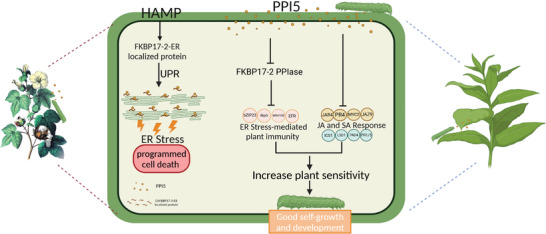
A simplified interaction model between PPI5 and GhFKBP17‐2 in modulating plant defense response and sensibility. Insect feeding induces HAMP, which activates the endoplasmic reticulum (ER) localization protein FKBP17‐2. This activation triggers the UPR in the ER, resulting in the induction of ER stress ultimately leading to PCD. In addition, the oral secretory protein PPI5 from *H. armigera* is introduced into the plant through mechanical wounds caused by feeding. On the one hand, PPI5 interacts with GhFKBP17‐2, inhibiting protein expression and PPIase activity, which suppresses ER stress‐mediated plant immunity. On the other hand, PPI5 also inhibits the JA and SA defense responses. This PPI5‐mediated inhibition of plant immunity may contribute to making the plant more susceptible to *H. armigera* invasion, allowing for improved feeding and development of the bollworm.

During the invasion of *H. armigera* on the plant, its chewing mouthparts cause severe mechanical damage to the plant. Through the wounds, *H. armigera* secretes the effector PPI5 into the plant and regulates the plant's immunity. The cotton bollworm *PPI5* is recognized by the plant as a pathogen‐associated molecular pattern (PAMP), activating the *FKBP17‐2‐*mediated ER stress response, which results in PCD in response to ER stress. Subsequently, PPI5 is secreted into the plant ER, where it targets the ER protein FKBP17‐2. FKBP17‐2 processes and folds PPI5, which is then degraded by PPI5 or inhibited by PPI5‐mediated expression, restoring ER homeostasis and suppressing ER‐mediated plant immunity. Furthermore, PPI5 enables *H. armigera* to colonize the plant by inhibiting the JA response in cotton and the JA and SA pathways in tobacco. Interestingly, the mode of action of PPI5 in enhancing susceptibility differs between cotton and tobacco. The transgenic tobacco lines *PPI‐1* and *PPI‐11* showed inhibition of JA and SA levels and related genes only upon induction by the cotton bollworm, probably due to the simultaneous maintenance of a JA and SA tandem response in tobacco while maintaining its own growth and development (Figure 6A–E). The transgenic cotton line *PPI‐N* down‐regulated directly JA levels and related genes upon induction. It is noteworthy that the transcription of *FKBP17‐2* was upregulated in *PPI‐1* compared to WT, whereas it was repressed following cotton bollworm induction. In contrast, the transcription level of Gh*FKBP17‐2* was repressed in *PPI‐N* but upregulated after cotton bollworm induction. The expression of the JA‐associated genes was repressed in *TRV: GhFKBP17‐2* compared to WT. JA levels were repressed in *CR‐GhFKBP17‐1/3* and up‐regulated in *OE‐GhFKBP17‐1/3*. However, there was no significant change in the transcript levels of *GhJAZ3* in *CR‐GhFKBP17‐1/3* and *OE‐GhFKBP17‐1/3*, which may indicate that FKBP may regulate insect defense in a JA‐independent manner. We found that the transcription of *Gh_Sca005135G01* in *TRV: GhFKBP17‐2* cotton was not inhibited by *PPI5* (Figure S16B, Supporting Information). Both JA and SA levels in *PPI‐1* and *PPI‐11* tobacco were suppressed following bollworm infection. In contrast, JA of *PPI‐N* cotton was inhibited after bollworm induction, although there was no significant difference in SA (Figure 6). The aforementioned outcomes collectively suggest that *PPI5* may be implicated in the presence of additional targets engaged in disparate plant signaling pathways in tobacco and cotton. The combined inhibitory effects result in increased susceptibility to bollworm invasion, thereby providing bollworms with access to superior growing conditions and plant nutrients (**Figure** **13**).

Effectors have been extensively studied in a variety of insects, but most of these studies have focused on sap‐sucking pests such as aphids and whiteflies. In contrast, the only effector studies in chewing insects involved GOX, HARP1, and HAS1.^[^ ^33^, ^35^, ^36^ ^]^ Cyclophilic proteins are well‐conserved and important in biology. CYPs in insects have been reported to be involved in many physiological processes. *Trypanosoma cruzi* Cyclophilin19, *Drosophila* shu and *A. lucorum* Al106 play an important role in parasite growth, primary biogenesis, and adaptive amplification cycles.^[^ ^54^, ^55^, ^56^, ^92^ ^]^ PPI5 is highly conserved in Lepidoptera and the growth of *H. armigera* dsPPI5 in cotton is significantly inhibited (Figure 2J; Figure 4). *PPI5* induces plant susceptibility by secreting into the plant through mechanical wounds and inhibiting plant defenses (Figure 2F; Figure 6). These results suggest that *PPI5* plays a key role in the growth of the cotton bollworm and the regulation of plant defense. FKBPs belong to the immunophilin family, which are known both as the receptors for immunosuppressant drugs and as PPIases that catalyze rotation of prolyl bonds.^[^ ^93^, ^94^ ^]^ In plants *FBKPs* are involved in the regulation of stress responses, hormonal regulation, biotic, and abiotic stresses, such as *FKBP15‐2*, *GmCYP1*, *AtFKBP65*, *PaFKBP12 MdFKBP62a* and *MdFKBP65a/b*.^[^ ^43^, ^71^, ^72^, ^77^, ^84^ ^]^ Overexpression and knockout of *GhFKBP17‐2* transgenic material will hopefully provide new endogenous insect resistance breeding material for cotton.

Overall, our results provide important mechanistic insights into how *H. armigera* effector *PPI5* mediates defense responses to colonize and survive on plants. The discovery of new effectors and the elucidation of their mechanisms of action could be valuable for plant‐insect resistance breeding strategies. It could provide key genes for plant endogenous insect resistance and insect growth and development, which could lay a good biological basis for cotton insect resistance breeding and new research ideas for pest management strategies.

## 4 Experimental Section

### Insect Culture and Feeding Test

Cotton bollworm (*H. armigera*) eggs were obtained from Henan Jiyuan Baiyun Industry Co., Ltd., Henan, China. The larvae were reared as described by Mao et al.^[^ ^95^ ^]^ For the insect feeding assay, second‑ or third‐instar larvae of *H. armigera* were weighed individually to control for the same conditions at the beginning of the assay.

The fully expanded leaves of transgenic and control plants were detached for 3rd larvae of *H. armigera* feeding bioassays in a Petri dish (9.0 cm in diameter) containing moist filter paper.^[^ ^96^ ^]^ The Petri dish was maintained under laboratory conditions for insect rearing, and the leaves were replaced every day. The insect weight and size were recorded.

### *H. Armigera* Saliva Collection

Oral secretions (OSs) were collected from larvae (third to fifth instar) and kept on ice during collection and then stored at −70 °C until use. Plants used for the experiment were 30 days old and the third leaf from the top of each plant was selected for treatment as described.^[^ ^97^ ^]^

### LC‐MS/MS Analysis for Unique Peptides of PPI5

To determine the protein in the oral cavity of *H. armigera* larvae, the third‐instar larvae were fed on cotton leaves for at least 24 h. The OS protein samples were frozen in liquid nitrogen and grounded with a pestle and mortar. After precipitated by TCA/Acetone buffer, SDT buffer (4% SDS, 100 mm Tris‐HCl, pH 7.6) was added. The mixture was precipitated at −20 °C for 4 h, centrifuged at 14 000 × g for 30 min at 4 °C, and the supernatant was discarded. Precooled acetone was added and washed three times. The precipitate was dried in the fume hood. A 30× volume (m/v) of SDT lysate was added and the precipitate was resuspended in Votex, followed by a boiling water bath for 10 min. Ultrasonic disruption (100 W, 10s on time, 10s off time, 10 cycles), boiling water bath for 10 min. After centrifugation at 14000 g for 30 min, the supernatant was removed and filtered through a 0.22 µm filter membrane to collect the filtrate. Protein quantification was performed by the BCA method. The samples were divided and stored at −80°C.

The reducing agent DTT (final concentration 100 mm) was added to 100ug protein solution respectively, boiled for 5 min, and cooled to room temperature. 200 µL of UA buffer was added and mixed well, transferred to a 30 kD ultrafiltration centrifuge tube, centrifuged at 14 000 g for 15 min, and the filtrate was discarded (repeat this step once). Then 100 µL of IAA buffer (100 mm IAA in UA) was added, shaken at 600 rpm for 1 min, and the reaction was carried out for 30 min at room temperature, protected from light, and centrifuged at 14 000 g for 15 min. A total of 100 µL of UA Buffer was added and centrifuged at 14 000 g for 15 min. Repeat this step twice. 100 µL of 25 mm NH_4_HCO_3_ solution was added and centrifuged at 14000 g for 15 min and repeated twice. 40 uL Trypsin Buffer (0.665, lug Trypsin in 40 uL 100 mm NH4HCO3) was added, shaken at 600 rpm for 1 min, and left at 37 °C for 16–18 h. Replace with a new collection tube and centrifuge at 14000 g for 15 min; 40 µL of 25 mm NH4HCO3 was added and centrifuged at 14 000 g for 15 min. The filtrate was collected. The peptides were removed using a C18 cartridge.

The enzymolysis products were injected on a Thermo Scientific Q‐Exactive HF‐ X mass spectrometer connected to an Easy‐nLC 1200 chromatography system (Thermo Scientific). The samples were transferred to Thermo scientific EASYcolumn (2cm*100 µm 5µm‐C18) and separated by Thermo scientific EASY column (75µm*100mm 3µm‐C18). Peptides were separated by chromatography and analyzed by mass spectrometry on a Q‐Exactive mass spectrometer (Thermo Scientific). The raw files were retrieved through MaxQuant software. The protein database was uniprot_Helicoverpa_armigera_10555_20200214.fasta.

### Complementary DNA (cDNA) Cloning and Sequence Analysis

Total RNA was isolated from the 4th instar nymph of *H. armigera* using RNAiso Plus reagent (Takara), following the manufacturer's instructions. Then, 3 µg RNA was reverse‐transcribed into cDNA using the PrimeScriptRT Reagent Kit with genomic DNA Eraser (Takara). This cDNA was used as a template to amplify the full length of the PPI5‐depleted signal peptide with the corresponding primers (Table S1, Supporting Information). Subsequently, the PCR product was gel‐purified (Promega), ligated into the pEASY‐T1 vector (TransGen), and sequenced. Finally, the amino acid sequence and protein functional domains of PPI5 were predicted by the National Library of Medicine (https://www.ncbi.nlm.nih.gov/cdd), UniProt (https://www.uniprot.org/), ExPASy Translate tool (http://web.expasy.org/translate/) and SMART software (http://smart.embl.de/), respectively.

### RNAi in *H. armigera* by injection of in vitro synthesized dsPPI5

A 407 bp fragment of the *PPI5* gene of *H. armigera* was amplified by PCR with primers containing a T7 promoter at each 5′end (Table S1, Supporting Information). Then, the PCR product was purified with a Gel Extraction kit (OMEGA) and used as templates for dsRNAs synthesis with the T7 RiboMAX Express RNAi System (Promega) according to the manufacturer's instructions. dsRNA against green fluorescent protein (GFP) was synthesized (dsGFP) and used as a control.^[^ ^98^ ^]^ 0.5 µg of dsRNA was injected into the *H. armigera* larvae on Day 1 of the 3rd instar by a micro‐injector (World Precision Instruments; Sarasota). At least 100 larvae were injected for each injection treatment. Furthermore, the developmental changes in body weight and body size were recorded four to six days after injection treatment. In total, 50–60 larvae were included per treatment and control. Finally, to determine silencing efficiency, the relative expression levels of PPI5 were measured at 24, and 48 h (*n  =*  5) using qRT‐PCR. β‐actin was used as an endogenous control to normalize the target gene expression in different experimental conditions. For each gene expression assay, three independent biological replicates were performed, and the data were reported as the mean ± SD/SEM. The QRT primers were listed in Table S1, Supporting Information.

### Plant Materials, Culture, and Treatment

Cotton (*Gossypium hirsutum*) and tobacco (*Nicotiana tabacum*, *Nicotiana benthamiana*) were grown in light incubators (22 °C, long day (LD), 16 h: 8 h, light: dark). When cotton was grown to the five‐leaf stage and *Nicotiana tabacum* leaves to seven, they were used to feed *H. armigera*. Treated leaves from three individual plants were harvested as one experimental replicate, flash‐frozen in liquid nitrogen, and stored at −80 °C.

### Plant Vector Construction and Plant Genetic Transformation

The CDSs of *PPI5* and *GhFKBP‐17‐2* were each cloned into the vectors pGWB405 and pGWB417. The CDS of *GhFKBP‐17‐2* was also cloned into the vector pRGEB32. The primers were listed in Table S1, Supporting Information. The recombinant vectors pGWB405‐PPI5, pGWB417‐GhFKBP‐17‐2, and pRGEB32‐GhFKBP‐17‐2 were electroporated into *Agrobacterium tumefaciens* GV3101. The genetic transformation method was described previously.^[^ ^99^, ^100^, ^101^ ^]^ All the sequences of primers used in the vector construction were listed in Table S1, Supporting Information.

### Molecular Analysis of Transgenic Plants

The young leaves of transgenic and wild‐type plants were selected for genomic DNA extraction using the Plant Genomic DNA Kit (Tiangen Biotech) and then used for PCR analysis. The specific primers for PCR detection in *PPI5* transgenic plants were listed in Table S1, Supporting Information.

To determine the expression level of *PPI5* in transgenic plants, total RNA was extracted from T1‐positive transgenic plants, using the RNAprep Pure Plant Kit (Cat. #DP441, TIANGEN), then 3 µg of total RNA was reverse transcribed to cDNA using the SuperScript III reverse transcriptase (Cat. No. 18080–093, Invitrogen). qRT‐PCR analysis was performed to determine gene expression levels, as described previously.^[^ ^96^ ^]^ The primers were listed in Table S1, Supporting Information.

### Sequence Analysis and Phylogenetic Analyses

The amino acid sequence of the *GhFKBP* family was BLAST searched on NCBI, and MGEA11 was used to construct an evolutionary tree. The amino acid sequence of PPI5 was BLAST searched on NCBI, and MGEA11 was used to construct an evolutionary tree of the top fifty insect species with high similarity as well as the reported *Apolygus lucorum* AI106, with PPI. The 233 proteins were analyzed using EffectorP (https://effectorp.csiro.au/) and TMHMM (http://www.cbs.dtu.dk/services/TMHMM). Signal peptide of PPI was convinced by SignalP 5.0 server (https://services.healthtech.dtu.dk/services/SignalP‐5.0/). The working model was painted using Biorender (https://app.biorender.com/). The image area was calculated using the ImageJ software.

### Analysis of Jasmonate (JA), Jasmonoyl‐l‐isoleucine (JA‐Ile), and Salicylic acid (SA) Contents in Plant Leaves

To measure the endogenous concentrations of JA, JA‐Ile, and SA, leaves of ≈100 mg were homogenized twice with 80% (v/v) cold methanol and shaken at 4 °C overnight in the dark. The dissolution, filtration, storage, and quantification of the combined extract were performed as described in Hu et al.^[^ ^102^ ^]^ To each sample were added 7.5 ng (±) of 9‐, 10‐dihydro‐JA (OlChemim), and 75 ng of 1‐naphthaleneacetic acid (Sigma‐Aldrich) as internal standards for the JA, JA‐Ile, and SA content assays. The test methods were described previously.^[^ ^103^ ^]^

### Subcellular Localization in Plant Leaves

Subcellular localization of proteins was analyzed both in the tobacco epidermis. The CDSs of *PPI5* and *GhFKBP‐17‐2* were each constructed into the vectors pMDC43, pGWB405, and pGWB454. The primers were listed in Table S1, Supporting Information. The recombinant vectors pGWB405‐PPI5, pGWB454‐PPI5 (RFP‐PPI5), and pGWB405‐GhFKBP‐17‐2 were electroporated into *Agrobacterium tumefaciens* GV3101. The GFP‐fusion proteins were transiently expressed in the tobacco epidermis as described previously.^[^ ^104^ ^]^ The localization of the proteins was observed using a Leica TCS SP2 confocal spectral microscope (Leica, Heidelberg, Germany). RFP‐ER was used as an ER marker.

### Prokaryotic Expression and Pull‐Down Assays

For prokaryotic expression assays, the CDS sequences of *GhFKBP17‐2*, *GhFKBP17‐2* mutants, and *GhFKBP* homologous genes were cloned into the vectors pGEX‐4T‐1 (Pharmacia) and purified from *Escherichia coli* BL21 (DE3), respectively. Recombinant GST fusion proteins were purified by Sangon Biotech GST‐Sefinose(TM) Resin 4FF (Settled Resin) (C600031). The experiment methods were described previously.^[^ ^105^ ^]^

For in vitro pull‐down assays, the CDS sequences of *PPI5* and *GhFKBP‐17‐2* were cloned into the vectors pGEX‐4T‐1 (Pharmacia) and PET‐28‐a (Novagen), respectively. The constructs His‐GhFKBP‐17‐2, His‐GFP‐PPI5, His‐GFP, and GST‐PPI5 were transformed into *E. coli* BL21 (DE3). Empty GST and recombinant GST‐ PPI5 proteins were used to pull‐down the His‐GhFKBP‐17‐2. The pull‐down proteins were purified with the MagneGSTTM Protein Purification System (Promega V8603) and MagneHisTM Protein Purification System (Promega V8550). The pull‐down assay was performed as described previously.^[^ ^106^ ^]^ The primers were listed in Table S1, Supporting Information. The antibodies of GFP, MYC, His, and GST were purchased from the ABclonal company. The anti‐PPI5 was synthesized in ABclonal. The antibody preparation process involved antigen preparation, animal immunization, and antibody purification. The anti‐PPI5 antibody was used in experiments with Japanese big‐eared white rabbits.

### Wounding Assay

The first pair of true leaves of the *G. hirsutum* plants were used in wounding treatments. For PPI5 treatments, recombinant proteins of His‐GFP and His‐GFP‐PPI5 were purified and dissolved in 20 mm Tris‐HCl buffer (pH 8.0) to a final concentration of 1 mg/ml. The wounded leaves were painted with indicated purified protein solutions.

To detect the translocation of prokaryotic‐expressed His‐GFP and His‐GFP‐PPI5 in plant cells, the second true leaves of cotton were punched and soaked into the 50 mm Tris hydrochloride buffer containing the purified histidine (HIS) fusion protein of His‐GFP and His‐GFP‐PPI5 (1.2 µg  µL^−1^) for 1 h. After washing with wash solutions (phosphate‐buffered saline containing 0.1% Tween 20) for three or four times to remove the extra proteins from the leaf surface. Confocal laser scanning microscopy was performed with an Olympus FV1200 microscope. Images were sequentially recorded with excitation wavelengths of 454 nm with the corresponding dichroic mirror and analyzed using CellSens Dimension (v.1.18; Olympus Corp., Beijing, China) software.

### Whole Amount Immunohistochemistry

The three‐week‐old cotton was incubated with fourth‐ instar larvae of *H. armigera*. The leaves after insect wounding damage were collected immediately and transferred to the FAA‐fixative solution for 4 h. The mechanically wounded leaves were used as control. Leaf samples were dehydrated through a series of graded alcohol solutions, followed by rehydration. After incubation for 2 h with blocking buffer (1XPBS containing 0.1% Tween 20 and 1% Albumin from bovine serum BSA), samples were incubated with the primary antibody (anti‐PPI5) at 4 °C overnight. The samples were washed by PBST (PBS containing 0.1% tween 20) for 4 times. The PPI5 signals were visualized by Digital Scanners (3DHISTECH). Product model: Pannoramic SCANII.

### LCI and BiFC Assay

For Bimolecular Fluorescence Complementation (BiFC) assays, the CDSs of *PPI5* and *GhFKBP17‐2* were respectively cloned into the vectors pXY104 and pXY106. For the Luciferase Complementation Imaging (LCI) assays, the CDSs of *PPI5* and *GhFKBP17‐2* were respectively cloned into the vectors JW771 and JW772.^[^ ^107^ ^]^ The recombinant vectors were transformed into *Agrobacterium tumefaciens* GV3101. YFP fluorescence in BiFC assays was observed using a Leica TCS SP2 confocal spectral Microsystems laser‐scanning microscope. LUC luminescence in LCI assays was observed using a CCD camera (Lumazome PyLoN 2048B).^[^ ^108^ ^]^

### Yeast Two‐hybrid Assay

The Matchmaker Gold Yeast Two‐Hybrid system (Cat. No. 630489) was used in (Yeast‐two‐hybrid) Y2H assays. The CDSs of *PPI5* were each constructed into yeast vector pGBKT7 (TaKaRa) and transformed into yeast strain Y2H. The full‐length CDSs of *GhFKBP‐17‐2* were cloned into the vector pGADT7, and introduced into yeast strain Y187. Interactions between different proteins were identified as growth on SD medium, SD‐Leu‐Trp (SD‐ TL), and SD‐Leu‐Trp‐His‐Ade (SD‐TLHA) (with X‐α‐Gal), respectively. The primers were listed in Table S1, Supporting Information.

### Virus‐induced Gene Silencing

VIGS assays were performed as reported previously.^[^ ^109^, ^110^ ^]^ Gene fragments (300–500 bp) of *GhFKBP‐17‐2* (*Ghir_D08G019080*) from CDS regions were constructed to the vector pTRV2. The primers were listed in Table S1, Supporting Information. The vector constructs were introduced into *Agrobacterium tumefaciens* strain GV3101. The recombinant vector *TRV: GhFKBP‐17‐2* was injected into the cotyledons of wildtype cotton plants, Jin668. Plants were grown in controlled environment rooms at 25 °C with a 16 h light/8 h dark photoperiod. VIGS efficiency was determined two weeks after infiltration, the leaves were collected and frozen in liquid nitrogen for target gene expression analysis. The successfully silenced plants were used for subsequent drought stress treatments.

### Trypan Blue Staining

Trypan blue staining was carried out as follows: Samples were boiled in trypan blue solution (0.25 mg ml^−1^) for 10 min and stained at 25 °C for 12 h. Subsequently, the samples were destained in chloral hydrate (2.5 mg ml−1) for 2 d and then images were analyzed to show dead cells.

### Yeast Signal Sequence Trap System

The pSUC2T7M13ORI (pSUC2) vector, which contains a truncated invertase gene (SUC2) lacking both the initiation Met and SP, was used. PPI5^sp^ and Avr1b^sp^ were cloned into pSUC2, and then, pSUC2‐derived plasmids were transformed into yeast strain YTK12. They were then plated on CMD‐W (minus Trp) plates and YPRAA plates containing raffinose and lacking glucose. In addition, invertase activity was determined by the reduction in triphenyltetrazolium chloride (TTC) dye to insoluble, red‐colored triphenylformazan. The method was described previously.^[^ ^111^ ^]^ The primers were listed in Table S1, Supporting Information.

### PPIase Assays

Protein from the transgenic plants OE‐GhFKBP17‐1/3 and CR‐GhFKBP17‐1/3 was extracted in RIPA Lysis Buffer (P0013C) and PMSF (ST506). The proteins in the assay buffer (35 mm HEPES, 0.015% TritonX‐100, pH 8.0) were mixed with 5 mm succinyl‐Ala‐Leu‐Pro‐Phe‐paranitroanilide (#S8511, Sigma), and the mixture was incubated on ice for 10 min, and each sample was placed in a spectrophotometer precooled to 8 °C. Immediately after the addition of 10 mm alpha‐chymotrypsin (Cat. No. C3142; Sigma‐Aldrich) at 8 °C, the absorbance at 390 nm was recorded every second for 30 seconds. The method was described previously.^[^ ^43^, ^76^ ^]^

### Statistical Analysis

Statistical data were presented as means ± SD or SEM. Significant differentials were analyzed by one‐way ANOVA or Student's t‐test. The mapping followed with Prism 8 (GraphPad Software, San Diego, CA, USA). The image area was calculated using the ImageJ software. For each assay, at least three biological replicates were recorded for each data point, and two or three independent experiments were performed.

### Availability of Data and Materials

The authors declare that all data supporting the findings of this study were available within the article and its supplementary information files or from the corresponding author upon reasonable request. The raw data of *H. armigera* oral secretions qualitative proteomic were deposited in a public repository and could be explored at https://doi.org/10.6084/m9.figshare.25663290.v1. There were no custom scripts and software used other than those mentioned in the “MATERIALS AND METHODS” section.

### Accession Numbers

Sequence data from this article could be found in the GenBank/EMBL data libraries and CottonFGD (https://cottonfgd.net/) under accession numbers *Ghir_D08G019080.1* (*GhFKBP17‐2*), *Ghir_A08G018190.1* (*GhFKBP17‐2A*), *Ghir_D05G014750.1* (*GhFKBP17‐1*), *Ghir_D08G006560.1* (*GhFKBP20*) and *Ghir_D04G015200.1* (*GhFKBP19*). Sequence data of *XP_021198138.1* (*PPI5*) could be found in the NCBI (https://www.ncbi.nlm.nih.gov/).

## Conflict of Interest

The authors declare no conflict of interest.

## Author Contributions

S.J., H.N. and X.Z. designed the research; S.J., X.Z., and Y.W. oversaw The study and advised on experimental design; Y.W., C.Z., M.Z., X.L and G.C. performed experiments and data analysis; M.A. and A.H. analyzed the data; Y.W. wrote the paper; W.M., X.N., K.L., and S.J. contributed to the critically revising of the manuscript.

## Supporting information



Supporting Information

Supporting Information

## Data Availability

Research data are not shared.
