# Supplementary material for: Cotton Bollworm (H. armigera) Effector PPI5 Targets FKBP17‐2 to Inhibit ER Immunity and JA/SA Responses, Enhancing Insect Feeding
Source: Adv Sci (Weinh). 2024 Oct 1;11(44):2407826. doi: 10.1002/advs.202407826 (PMC11600268; doi:10.1002/advs.202407826)
Supplement: Supplementary file 1 — Supporting Information [file ADVS-11-2407826-s001.docx]

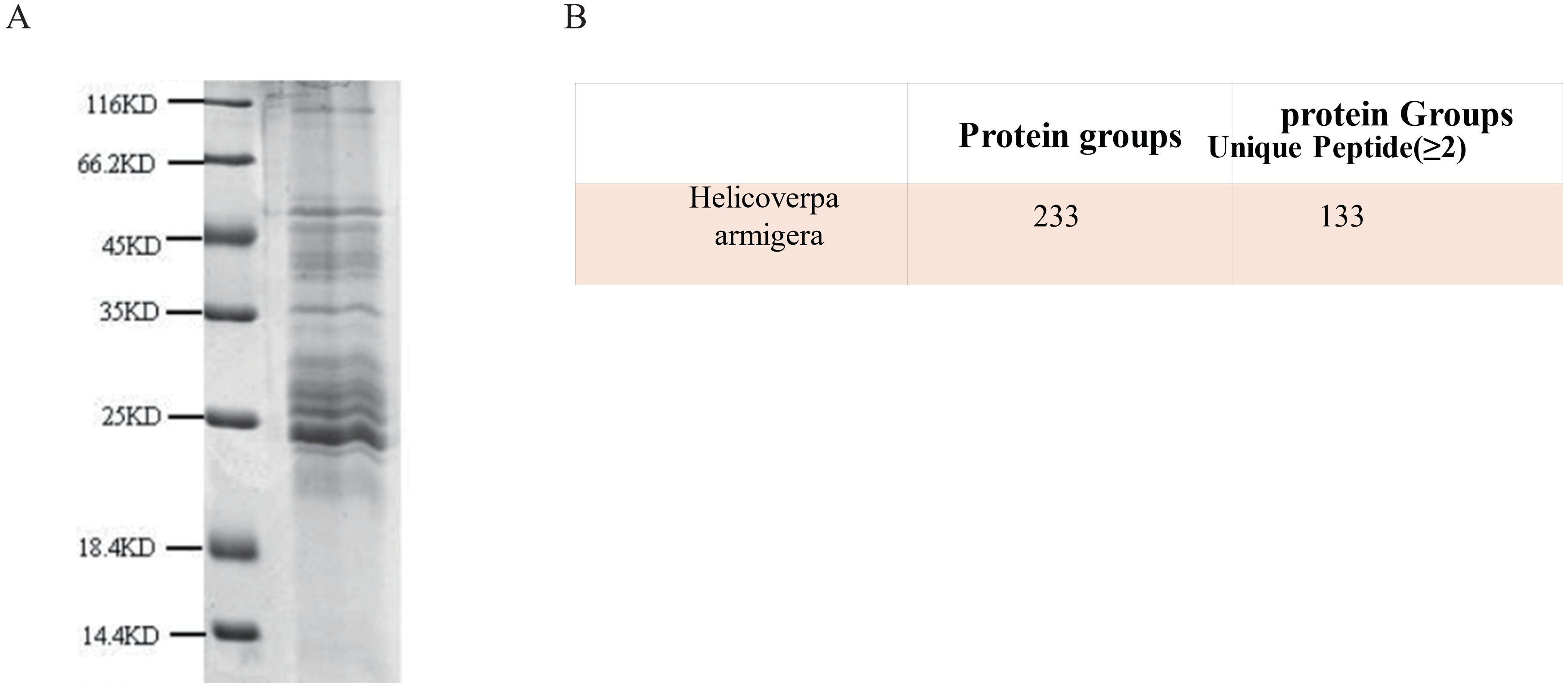


Figure S1. *H. armigera* oral protein identification results. A, Western blot detection of *H. armigera* oral proteins. B, *H. armigera* oral peptides and proteins identification results.


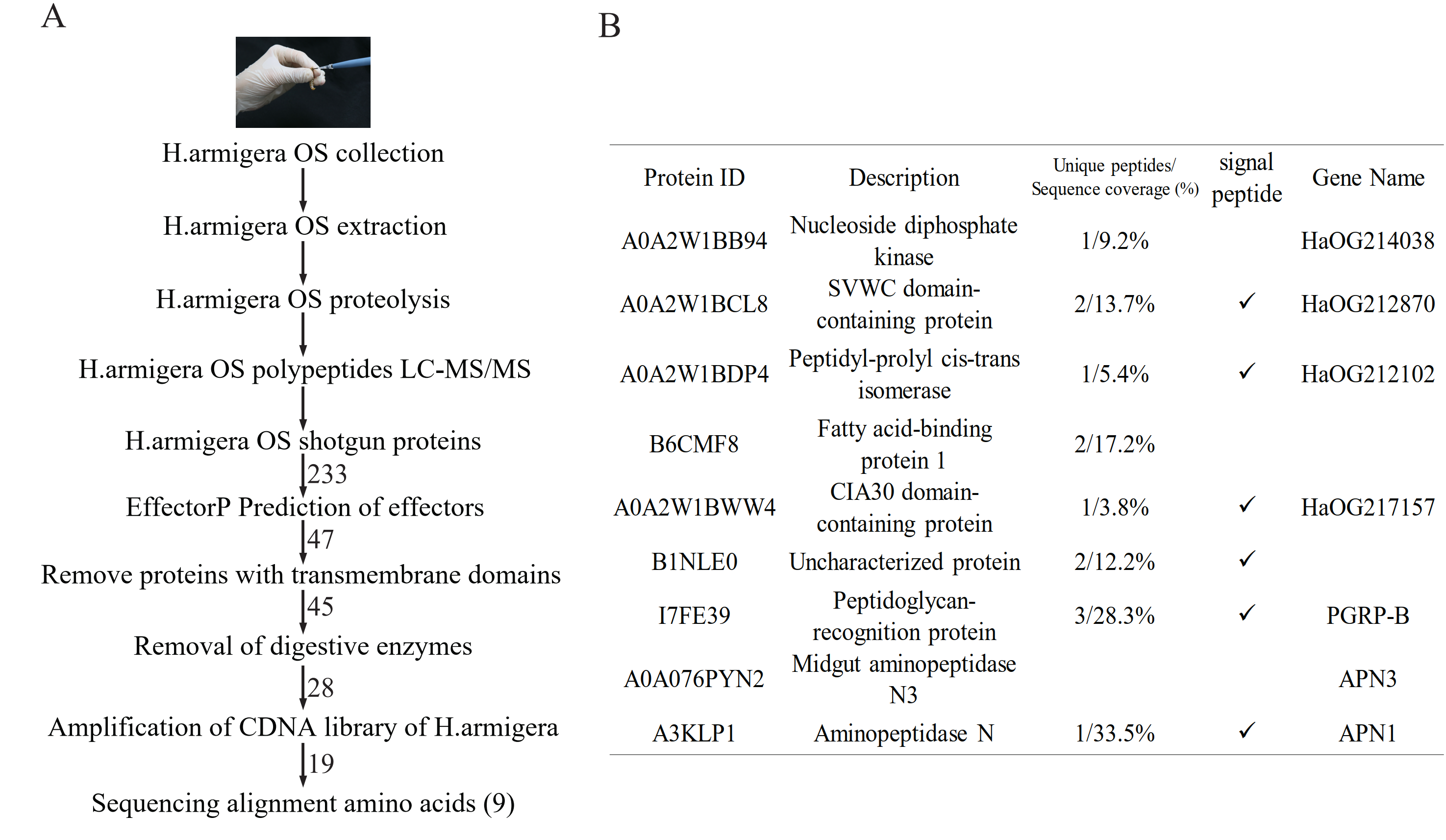


Figure S2. Screening of *H. armigera* candidate effectors. A, *H. armigera* oral secretions (OSs) collection and bioinformatic analysis for the identification of candidate effectors. B, Nine *H. armigera* candidate effect proteins.


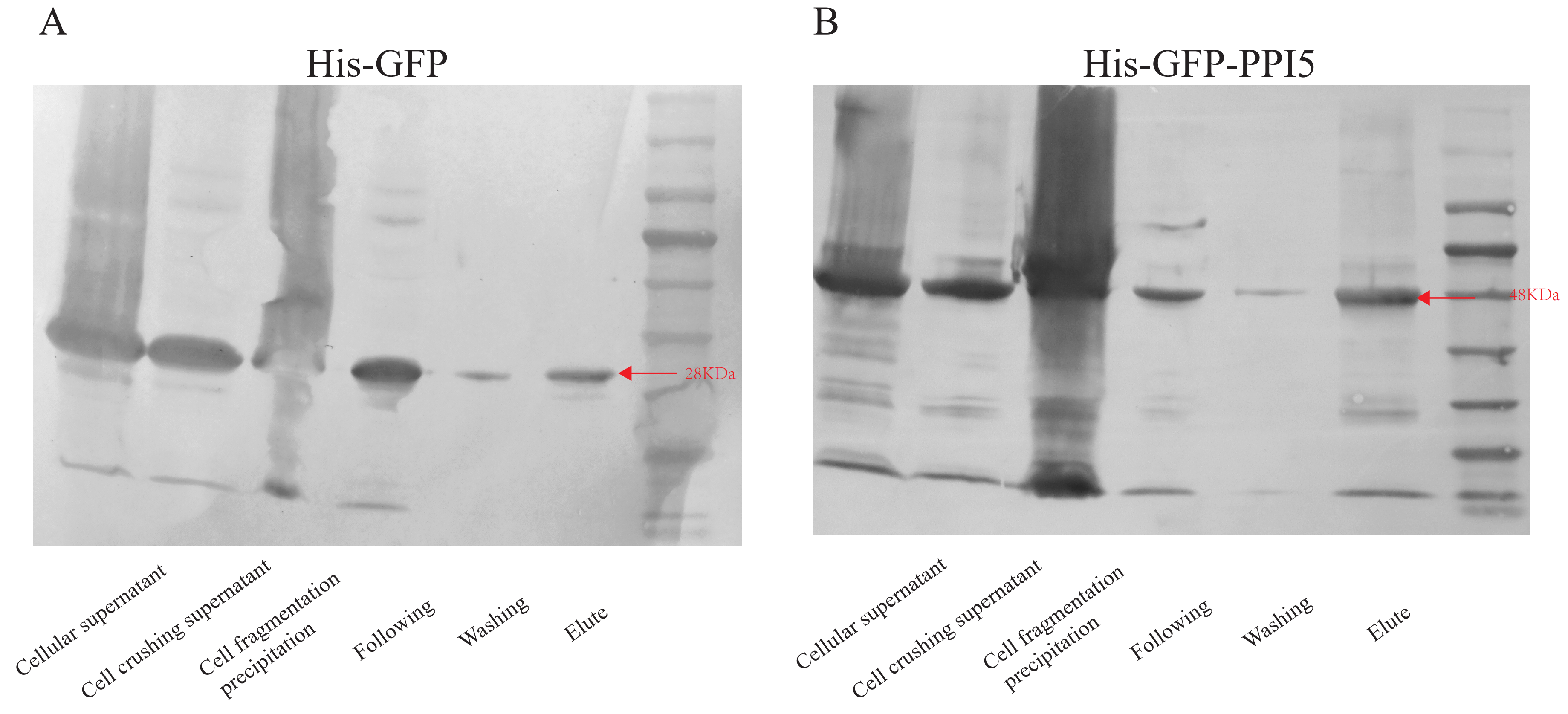


Figure S3. Western blot (WB) detection of purified fusion protein of His-GFP and His-GFP-PPI5. A-B, WB detection of His-GFP and His-GFP-PPI purified protein. AntiHIS antibody was used to detect. His-GFP has a molecular weight of 28KDa, and His-GFP-PPI has a molecular weight of 48KDa. The red arrows indicate the molecular weights of His-GFP and His-GFP-PPI5, respectively.


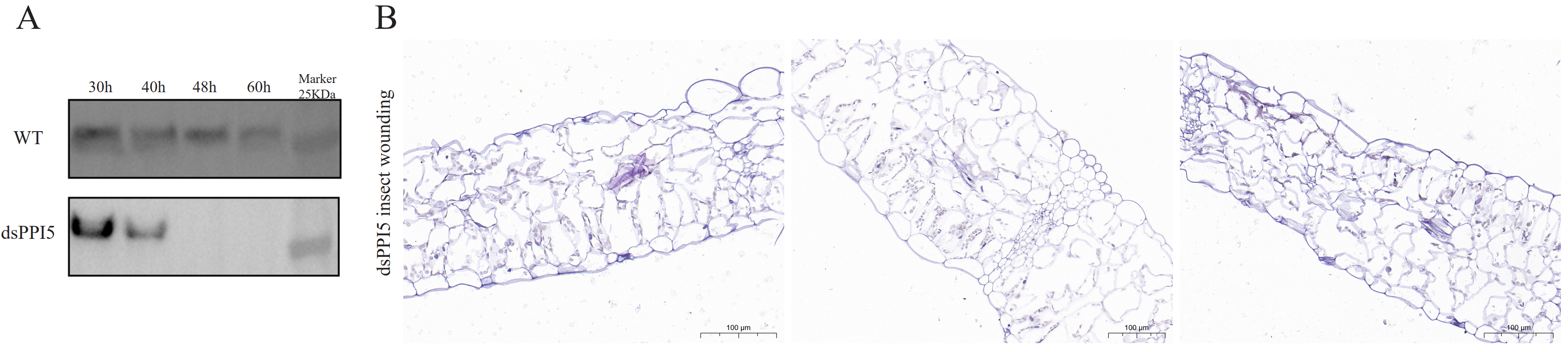


Figure S4. The polyclonal antibody to PPI5 is specific. A, Western blot detection of WT and dsPPI5 cotton bollworm proteins. B, Whole amount immunohistochemistry detection of dsPPI5 at the chewing sites of cotton leaves. Anti-PPI5 antibody was used to detect.


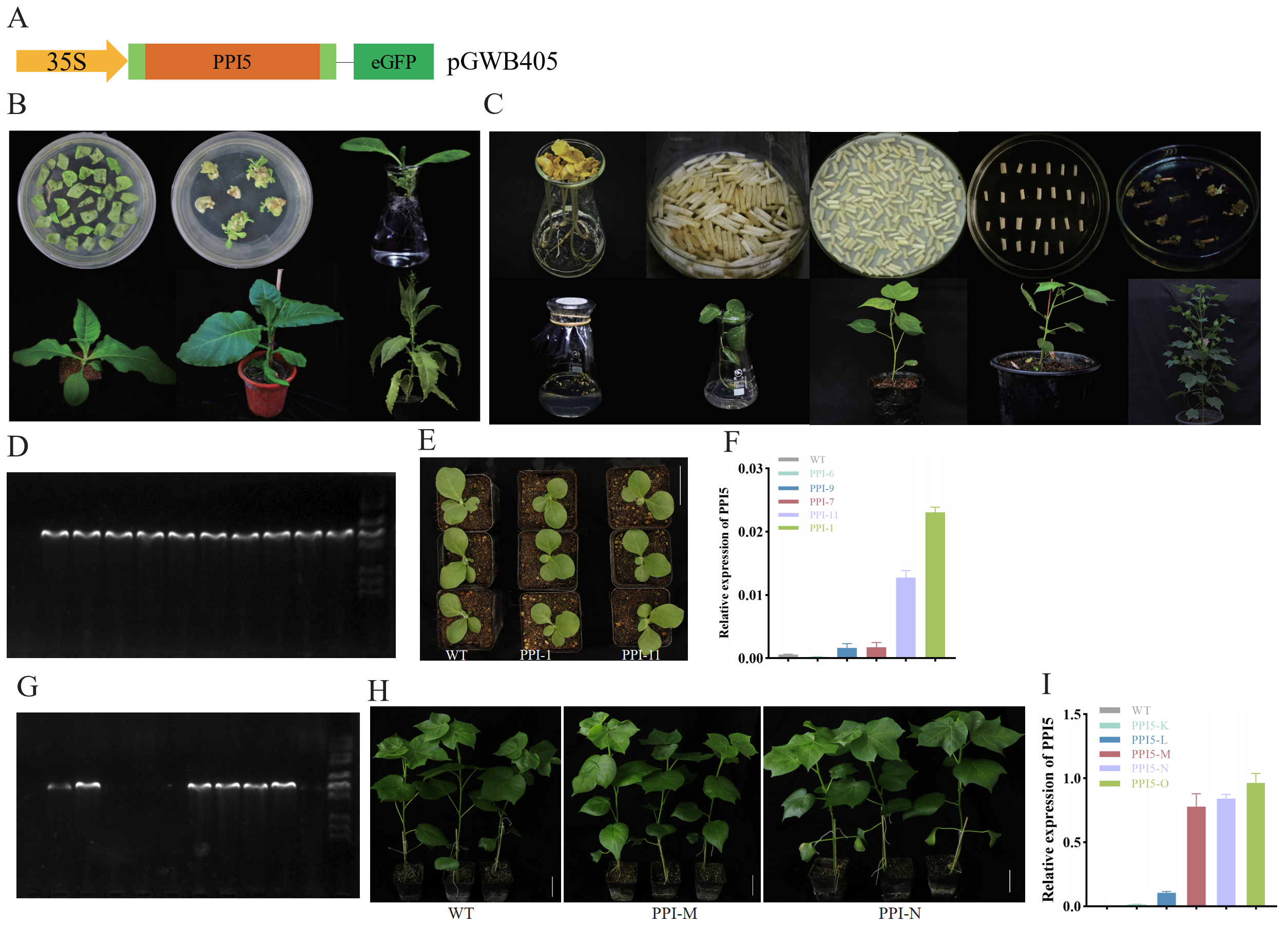


Figure S5. Overexpression of *PPI5* in cotton and tobacco. A, B, C. *PPI5* was constructed into PGWB405 vector containing 35s promoter and infected tobacco leaves and cotton hypocotyls by [*Agrobacterium*](https://en.wikipedia.org/wiki/Agrobacterium)-mediated genetic transformation. D, G. T1 generation tobacco and cotton were detected by PCR, respectively. E. Overexpression of *PPI5* in tobacco had no obvious visible growth and development phenotypes. Scale bar: 5cm. F. Transcriptional expressions of *PPI5* in transgenic tobacco. H. Overexpression of *PPI5* in cotton had no obvious visible growth and development phenotypes. Scale bar: 5cm. I. Transcriptional expressions of *PPI5* in transgenic cotton. The transcriptional expressions of *PPI5* were detected by qRT-PCR.


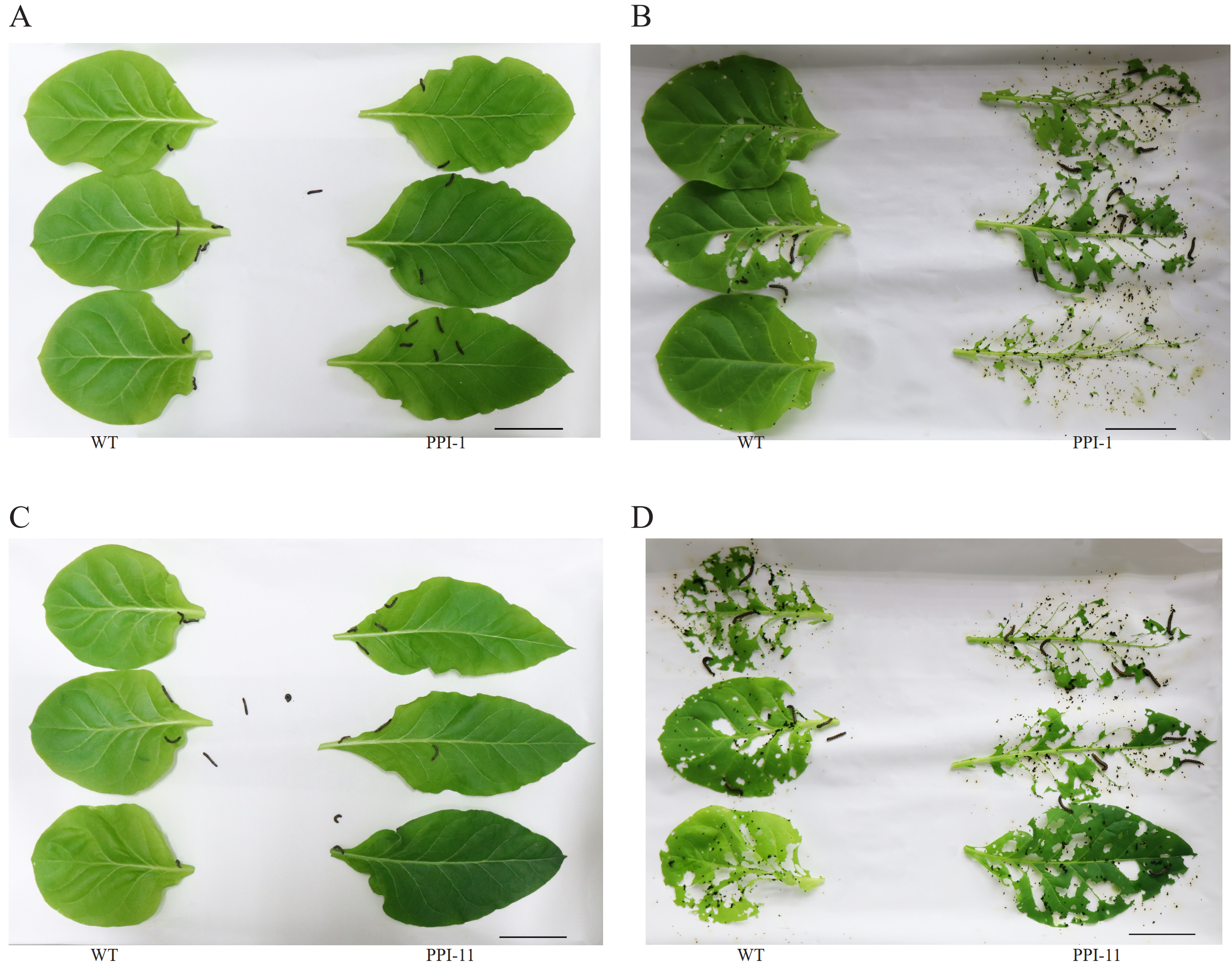


Figure S6. Cotton bollworm are more inclined to feed on *PPI-1* and *PPI-11* compared to WT tobacco. A-D, Preference feeding experiment of cotton bollworm. Twenty bollworm were randomly placed in the center of the tobacco and allowed to feed. After 24 hours, the distribution number of bollworm and the damaged area of leaf were counted.


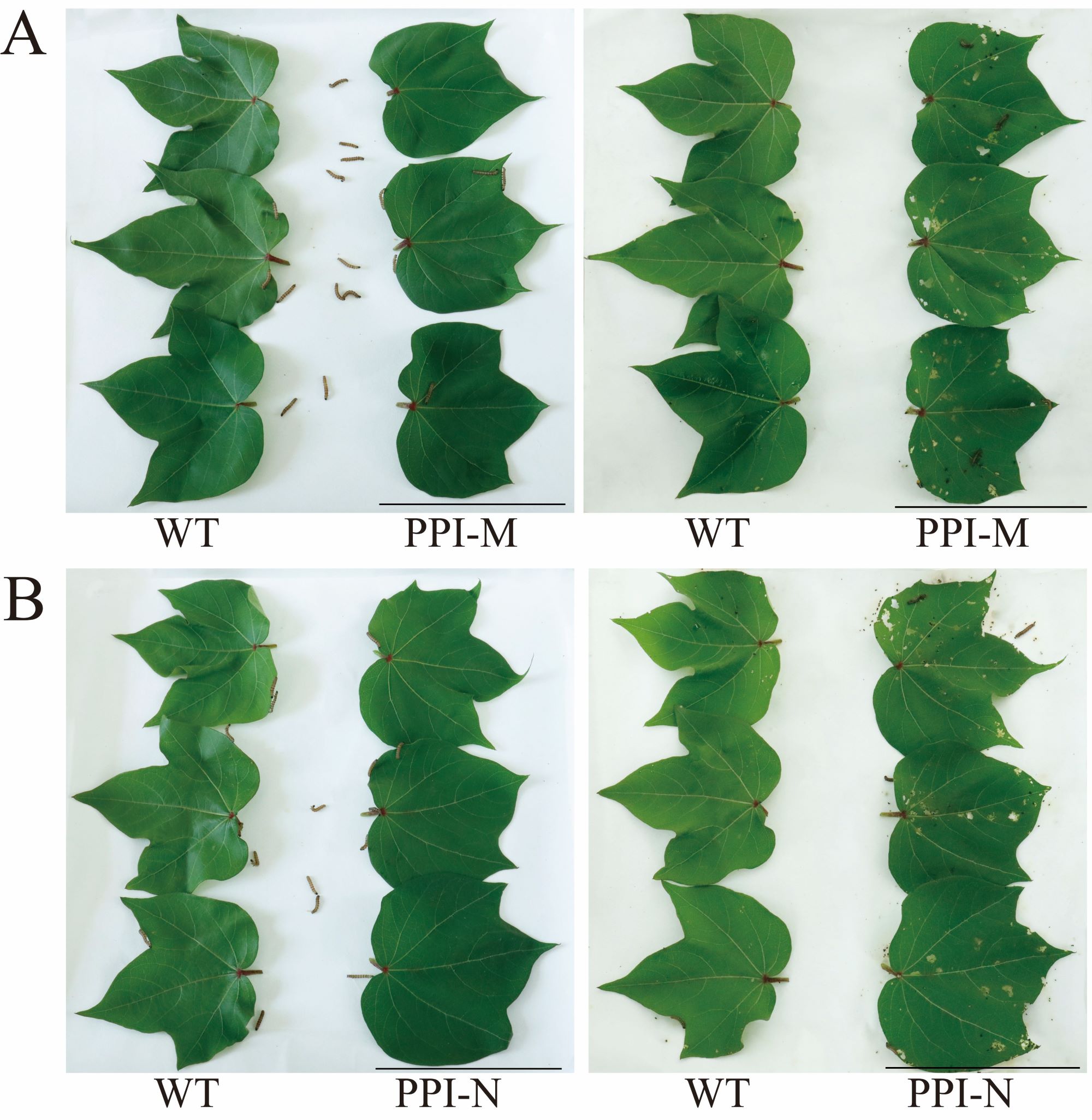


Figure S7. Preference feeding experiment of cotton bollworm in WT, *PPI-M* and *PPI-N* cotton. A-B, Sixteen bollworms were randomly placed in the center of cotton (WT, PPI-M and PPI-N) and allowed to feed. After 24 hours, the damaged area of leaf was counted. Scale bar: 5cm.


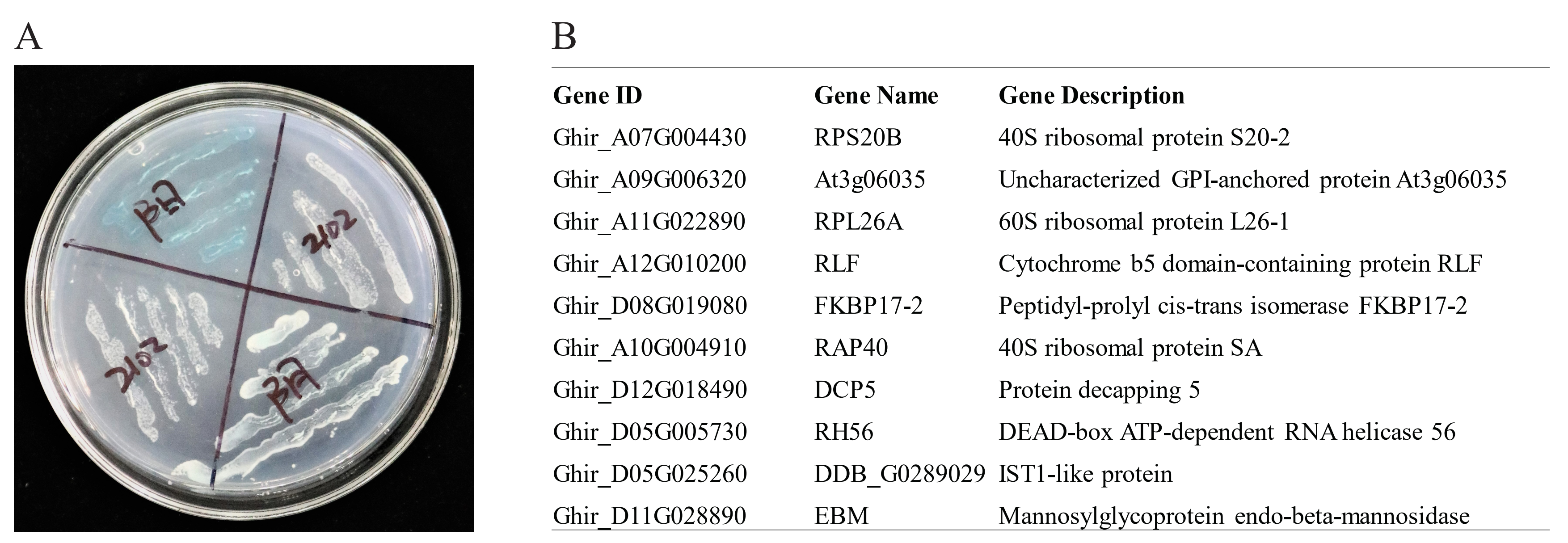


Supplemental Figure S8. Self-activation of PPI5 and screening of cotton yeast library. A, PPI5 has no self-activation activity. B, Initial identification of the possible interacted genes with PPI5 by screening of yeast library, PPI5 used as a binding domain. Those genes were able to grow on the SD-TLHA+X-α-gal (T: Trp; L: Leu; H: His; A: Ade) medium.


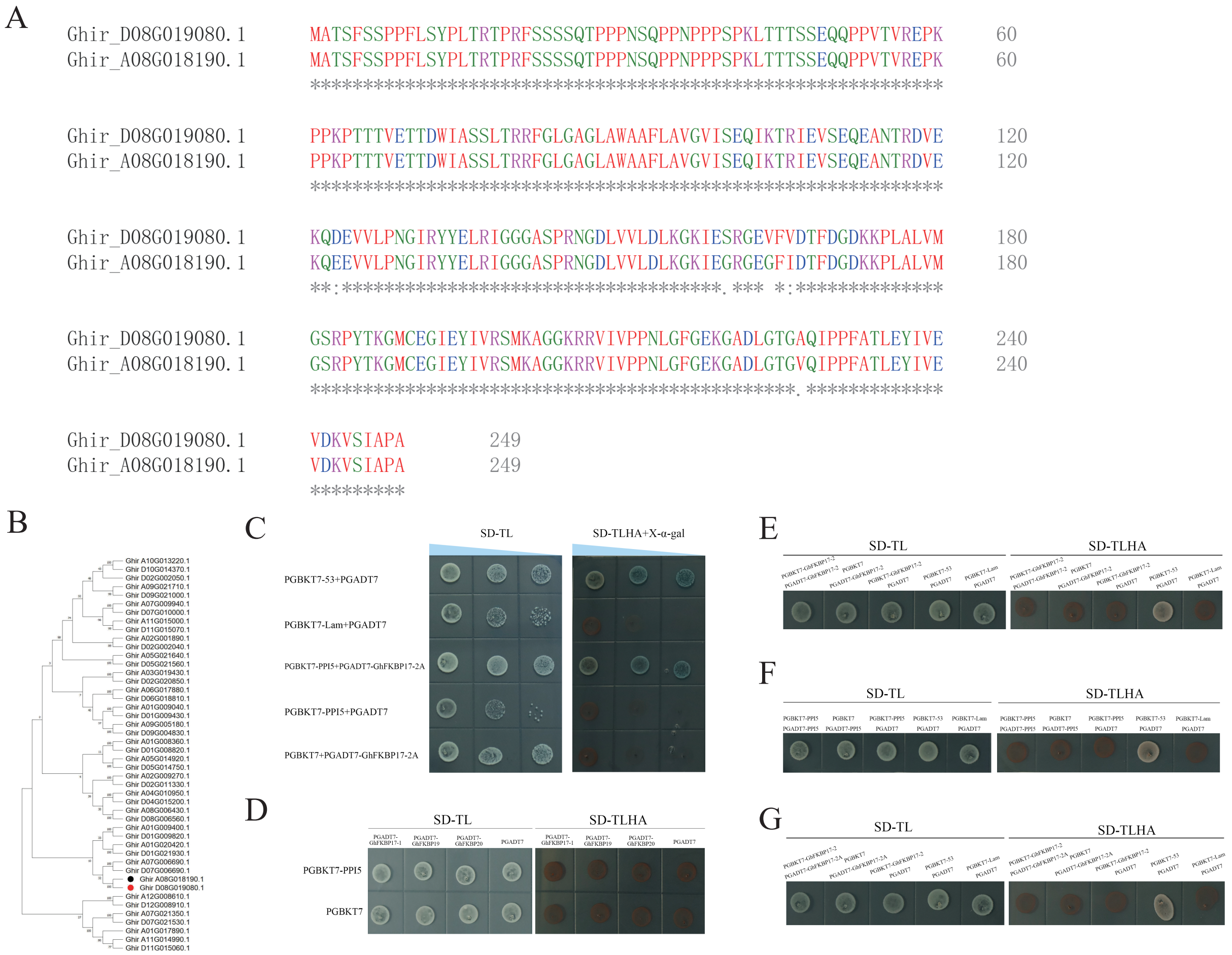


Figure S9. Comprehensive analysis of the *GhFKBP* gene family and PPI5. C, Amino acid sequence analysis of GhFKBP17-2 and GhFKBP17-2A. *GhFKBP17-2*: *Ghir_D08G019080.1*; *GhFKBP17-2A*: *Ghir_A08G018190.1*. D, Evolutionary tree analysis of GhFKBP gene family. E-F, Y2H assay to detect interaction between PPI5 and GhFKBP homologous genes. G-H, Y2H assay for detecting PPI5 and GhFKBP17-2 self-interaction. I, Y2H assay to detect interaction between GhFKBP17-2 and GhFKBP17-2A. Interactions were examined in SD-TLHA (SD-Leu-Trp-His-Ade). SD-TL: SD-Leu-Trp.


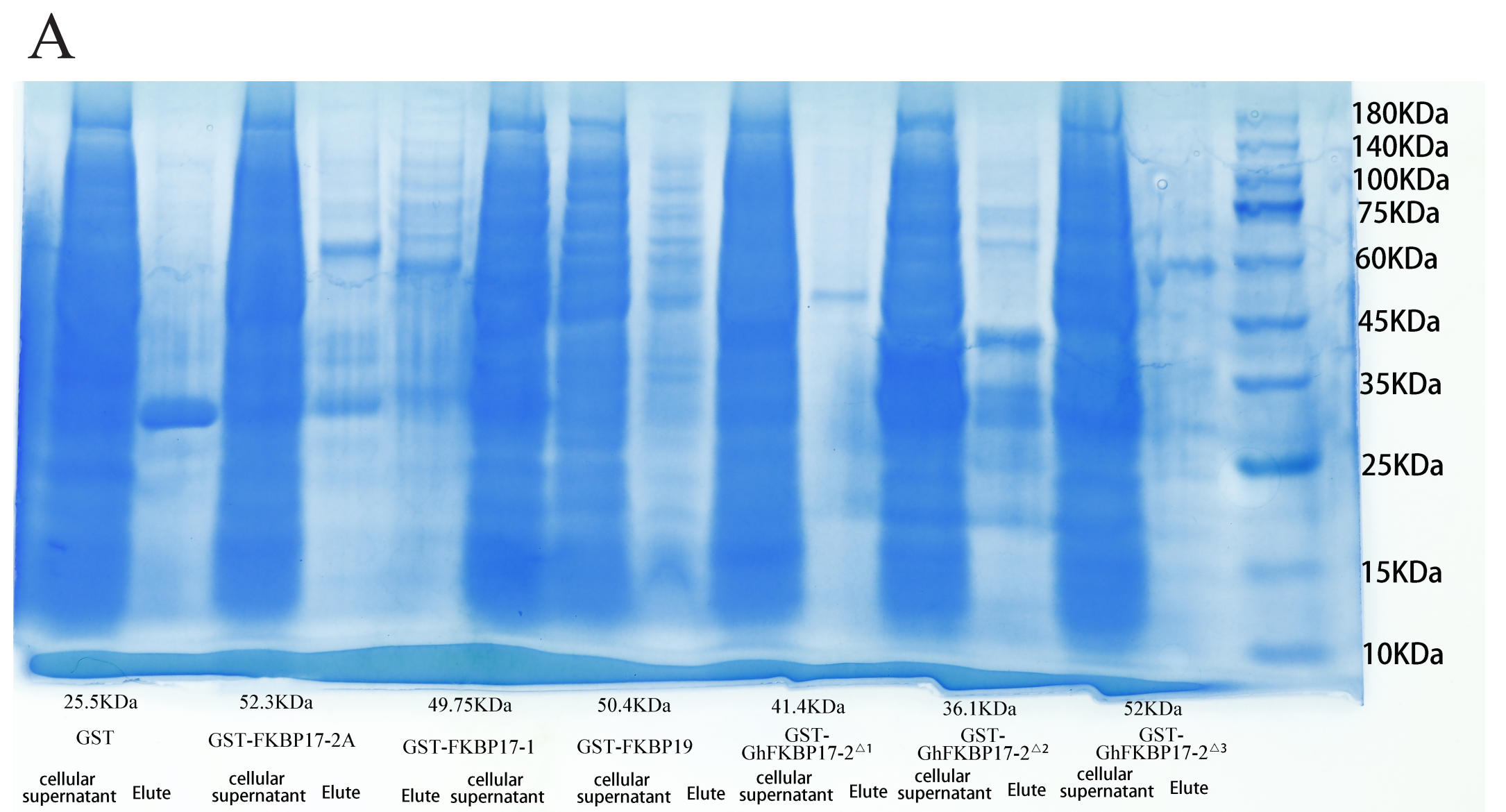


Figure S10. Molecular weight detection of *GhFKBP* homologous gene and *GhFKBP17-2* mutants. A, The prokaryotic expression protein supernatant and purified proteins (Elute) were detected by Coomassie brilliant blue staining. The molecular weight of GST is 25KDa. The molecular weight of GST- GhFKBP17-2A is 52.3KDa. The molecular weight of GST- GhFKBP17-1 is 49.75KDa. The molecular weight of GST-GhFKBP19 is 50.4KDa. The molecular weight of GST-GhFKBP17-2^△1^ is 41.4KDa. The molecular weight of GST-GhFKBP17-2^△2^ is 36.1KDa. The molecular weight of GST-GhFKBP17-2^△3^ is 52KDa.


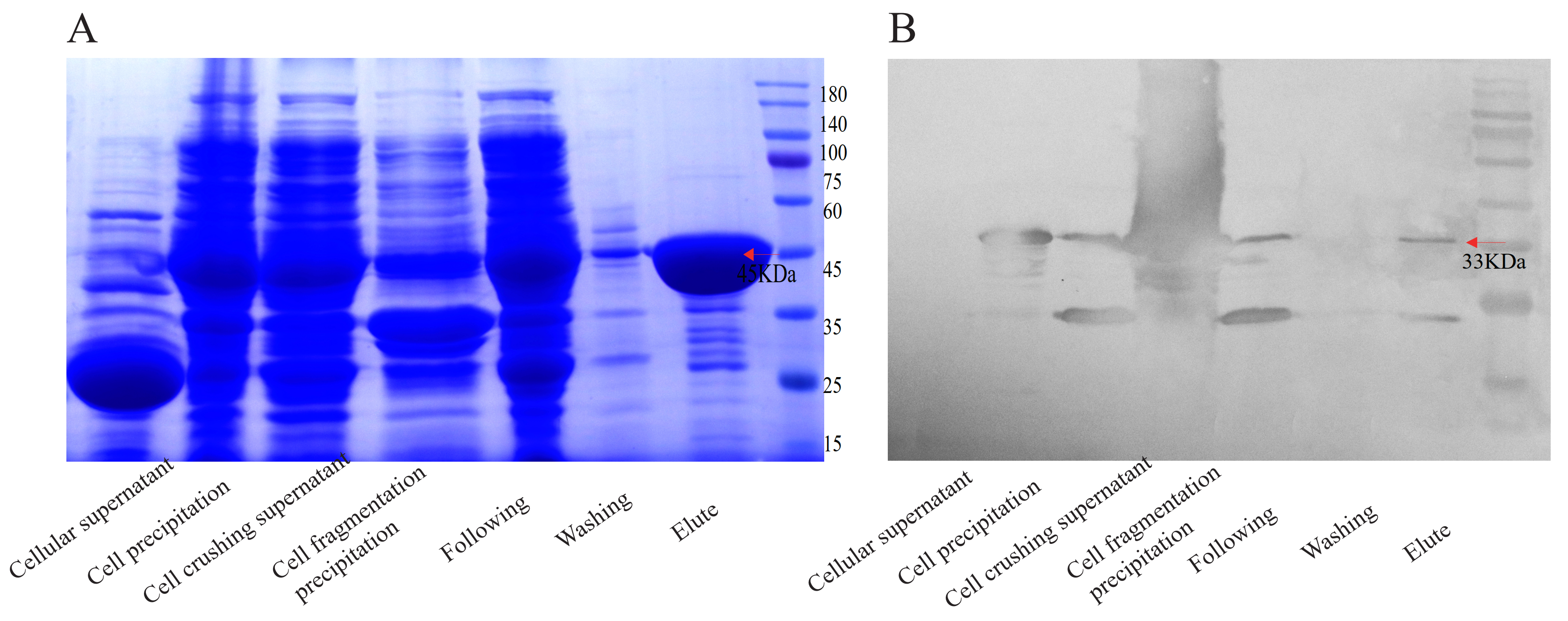


Figure S11. Molecular weight detection of purified proteins of GST-PPI5 and His-GhFKBP17-2. A, The purified protein of GST-PPI5 was detected by Coomassie brilliant blue staining. The molecular weight of GST-PPI5 is 45KDa. B, Western blot detection of purified protein of His-GhFKBP17-2. AntiHIS antibody was used to detect. The molecular weight of His-GhFKBP17-2 is 33KDa.


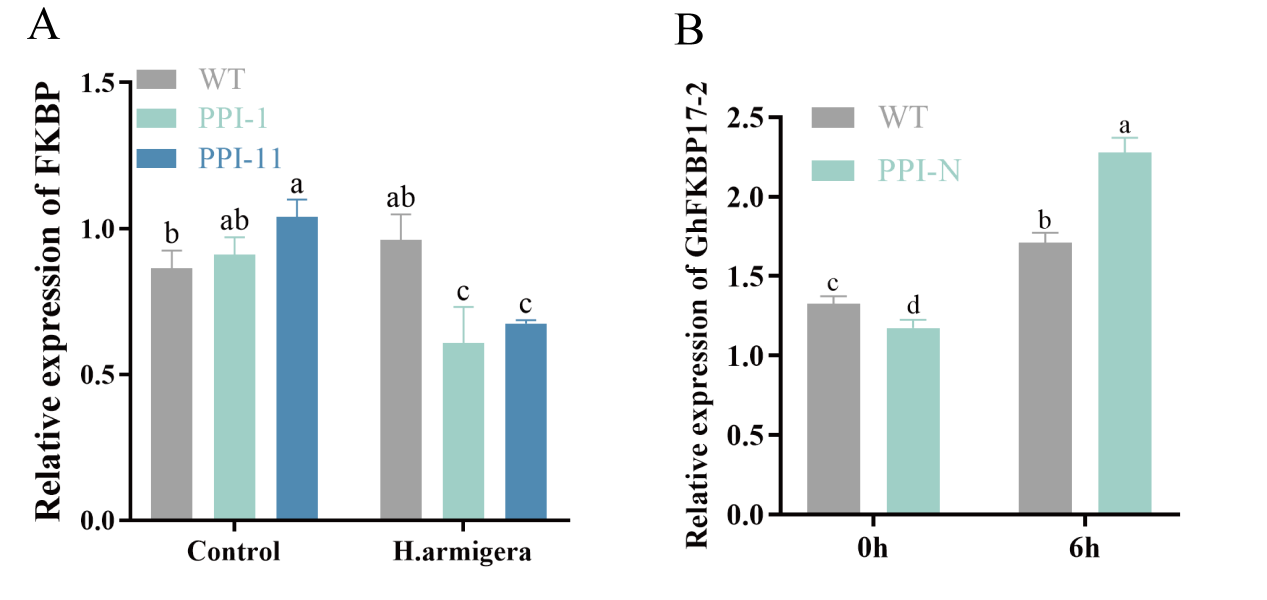


Figure S12. Transcriptional expressions of *FKBP* in *PPI-N* cotton and *PPI-1/11* tobacco. A, Transcriptional expressions of *FKBP* in *WT*, *PPI-1* and *PPI-11* tobacco. B, Expression levels of *GhFKBP17-2* in cotton. Cotton bollworm fed on cotton and tobacco plants for 6h. Young leaves with feeding traces were sampled, frozen in liquid nitrogen, and stored at -80 °C. The transcriptional expressions of gene were detected by qRT-PCR. Error bar, ±SD (n = 3 biological replicates). Significances were examined by one-way ANOVA, p<0.05.


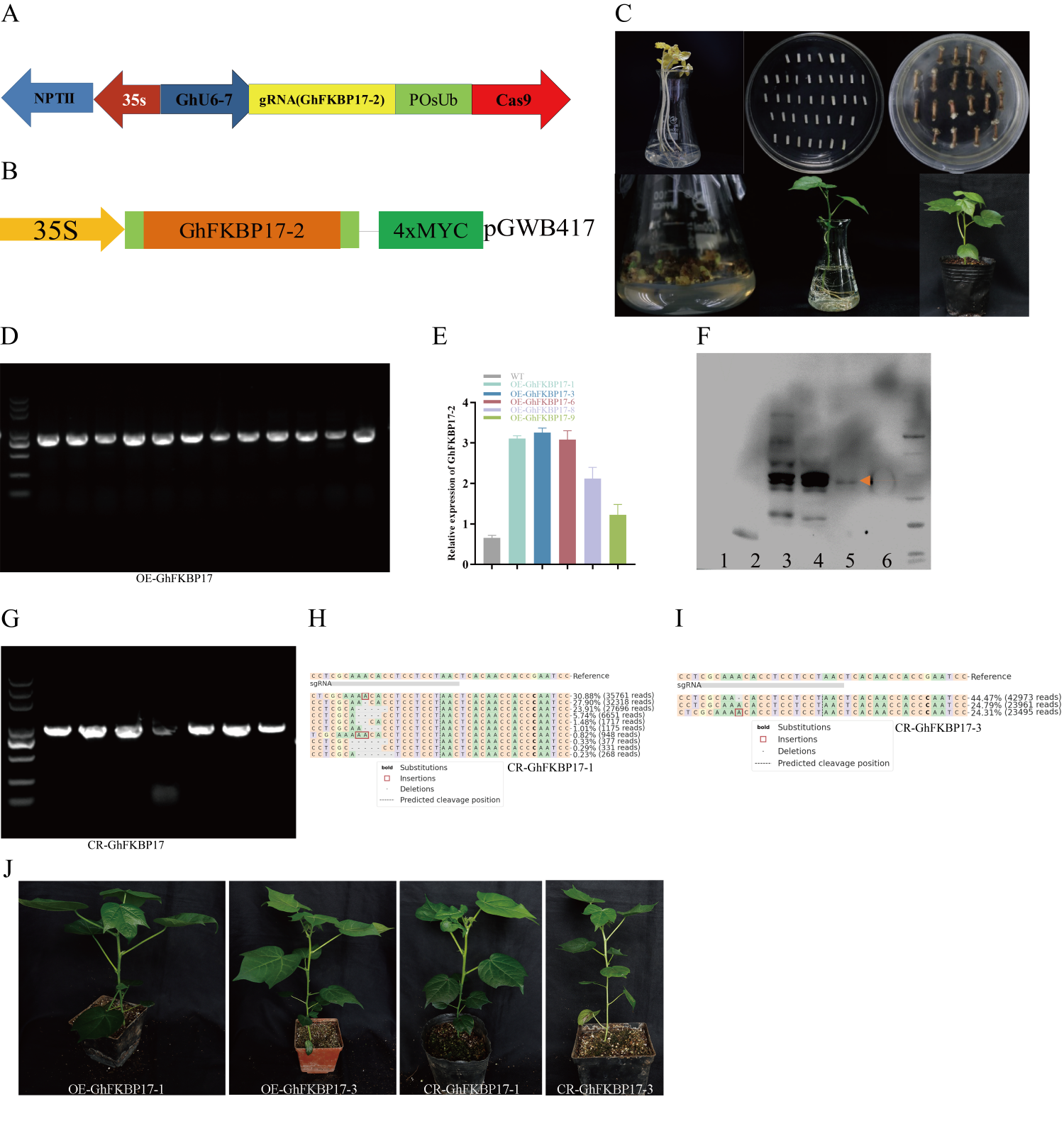


Figure S13. Molecular detection of transgenic *OE-GhFKBP17* and *CR-GhFKBP17* cotton. A, CRISPR/Cas9-mediated knockout vector for *GhFKBP17-2* in cotton. B, Overexpression vector containing 35S promoter for *GhFKBP17-2* (35S: GhFKBP17-2). C, *Agrobacterium*-mediated genetic transformation of cotton. D, G, PCR for transgenic overexpression and knockdown of cotton. E, Transcriptional levels of *GhFKBP17-2* in WT and transgenic overexpressed cotton. F, Immunoblotting analysis of in WT and five *OE-GhFKBP17* lines (1: WT; 2: *OE-GhFKBP17-9*; 3: *OE-GhFKBP17-1*; 4: *OE-GhFKBP17-3*; 5: *OE-GhFKBP17-6*; 6: *OE-GhFKBP17-8*). The molecular weight size of OE-GhFKBP is 36 KDa. AntiMYC antibody was used to detect. H, I, Editing efficiency and targeting sites of *CR-GhFKBP17-1* and *CR-GhFKBP17-3*. Gene expressions were detected by qRT-PCR. Error bar, ±SD (n = 3 biological replicates). J, *OE-GhFKBP17-1/3* and *CR-GhFKBP17-1/3* phenotypes. The transgenic plants had no obvious visible growth and development phenotypes.


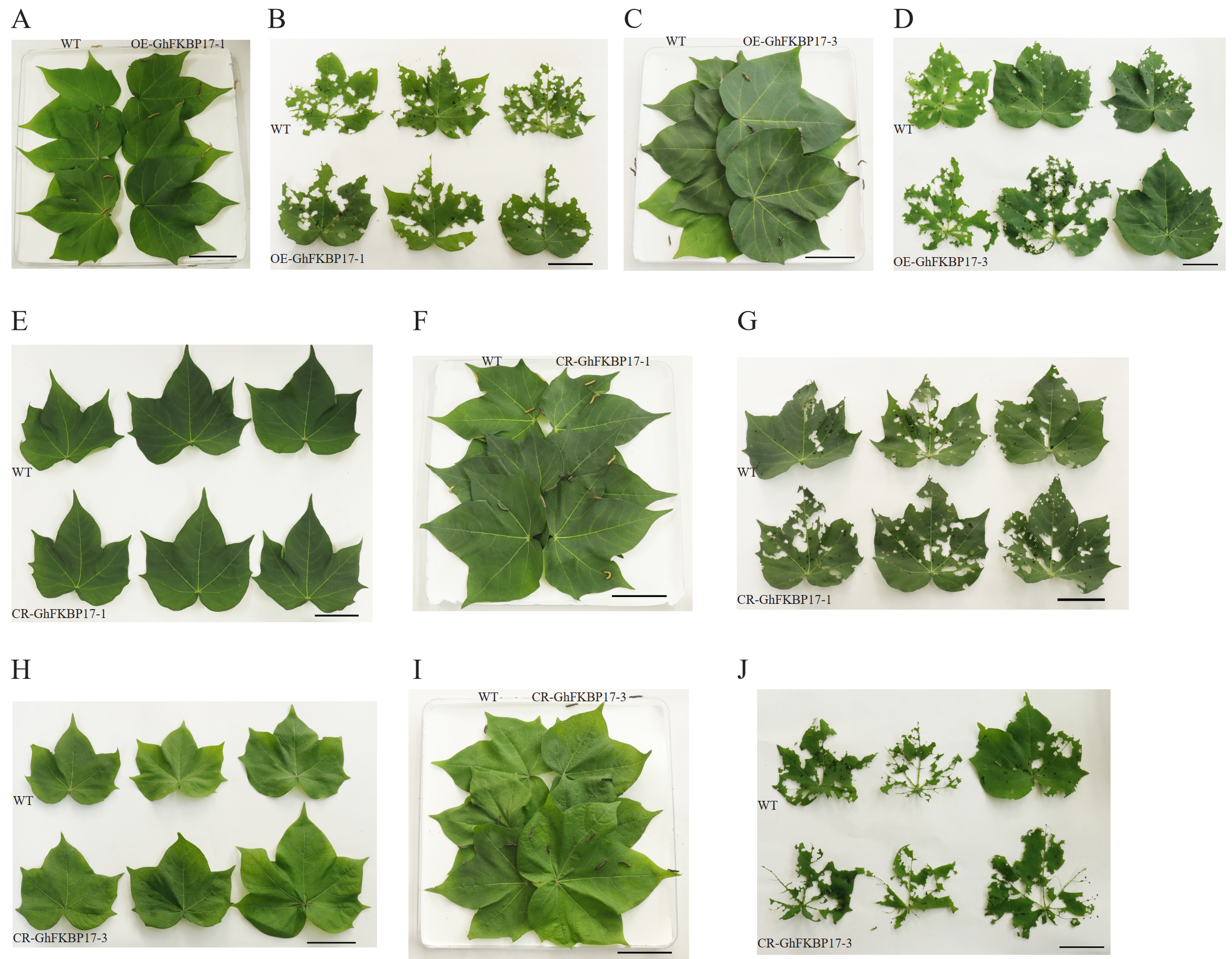


Figure S14. Preference feeding experiment of cotton bollworm on *OE-GhFKBP17-1/3* and *CR-GhFKBP17-1/3* cottons. A-D, Twenty cotton bollworms were randomly placed in the center of cotton (A-D, WT and *OE-GhFKBP17*; E-G, WT and *CR-GhFKBP17*) and allowed to feed. After 24 hours, the damaged area of leaf was counted. Scale bar: 5cm.


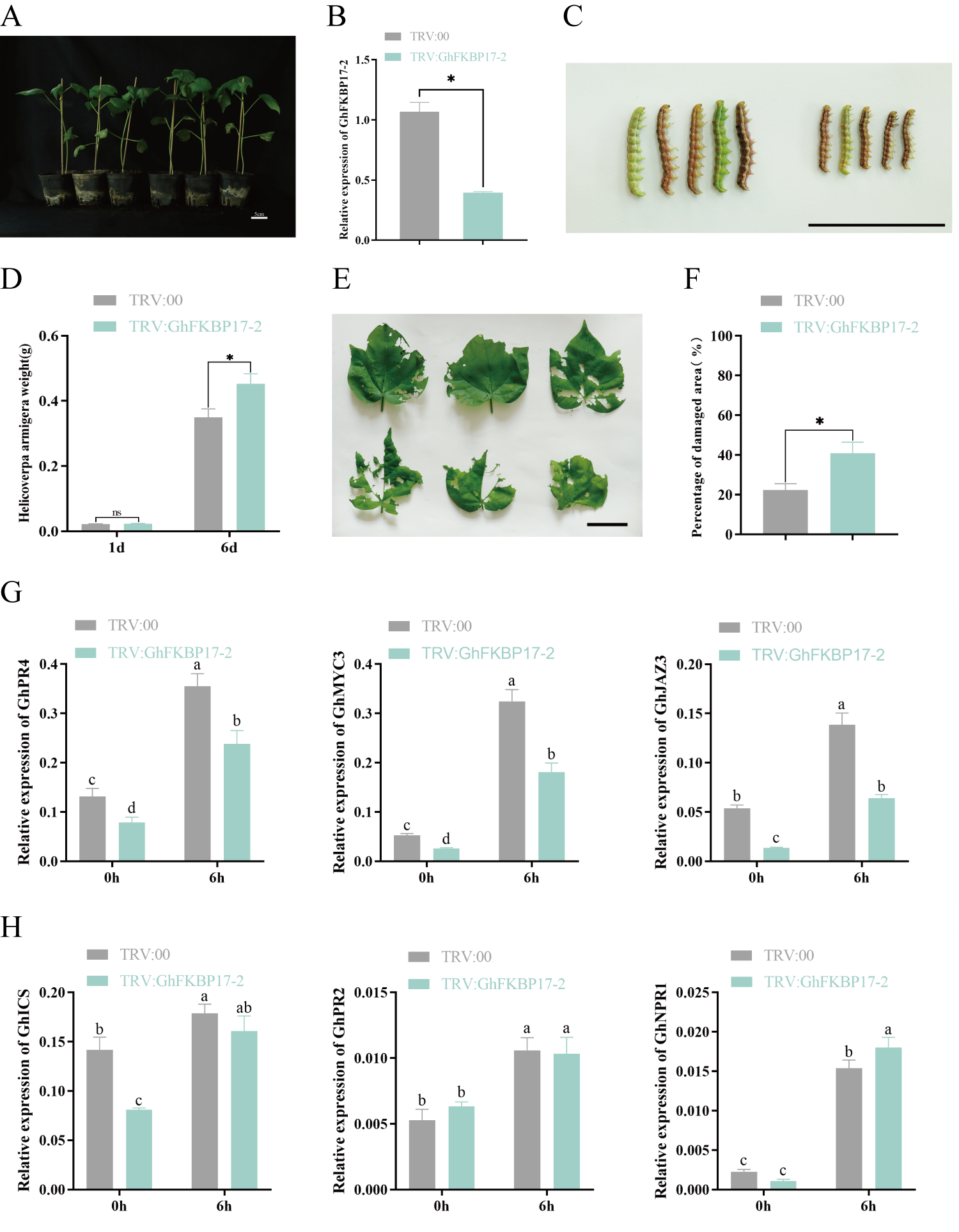


Figure S15. Expression of JA, SA related genes and the effects on feeding of cotton bollworm in *TRV:00* and *TRV: GhFKBP17-2* cotton. A, *GhFKBP17-2*-silenced plants had no obvious visible growth and development phenotypes. B, Relative expression levels of *GhFKBP17-2* in silenced and control plants. C-D, The body weight and their body size of cotton bollworm larvae fed on *TRV:00* and *TRV: GhFKBP17-2* cotton. E-F, Preference feeding experiment of cotton bollworm in cotton. F, The ratio of damaged area of leaf was counted. G, Transcriptional expressions of JA related genes in cotton. H, Transcriptional expressions of SA related genes in cotton. Error bar, ±SD (n = 3 biological replicates, 12-18 larvae were used as one replicate). Significances were examined by Student’s t test and one-way ANOVA. *P < 0.05. scale bars: 5cm.


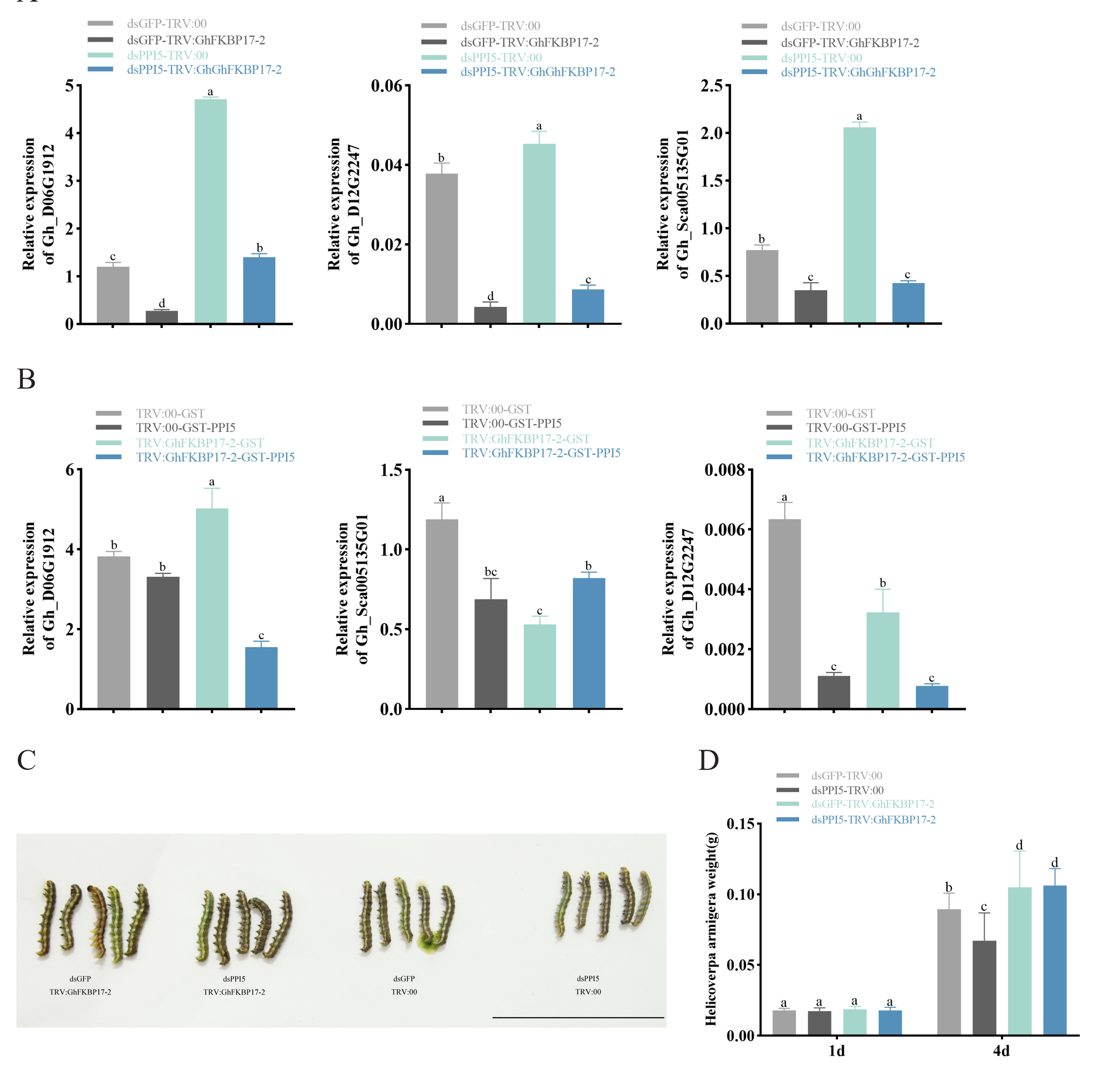


Figure S16. Wounding responses and insect resistance were not obviously affected by PPI5 in the *TRV: GhFKBP17-2* cotton. A-B, Transcriptional expression of proteinase inhibitor genes in the *TRV:00* and *TRV: GhFKBP17-2* cotton. A, Cotton leaves were induced by dsGFP and dsPPI5 cotton bollworm feeding for 4h B, Cotton leaves were mechanically wounded and painted with the prokaryotically expressed GST-PPI5 or GFP solutions (1 mg/mL) on the wounded sites. Samples were collected 4 h later, and the gene expressions were detected by qRT-PCR. Error bar, ±SD. C-D, The weight and body size statistics of dsGFP and dsPPI5 cotton bollworm larvae fed on *TRV:00* and *TRV: GhFKBP17-2* cotton. Error bar, ±SD (n = 3 biological replicates, 12-18 larvae were used as one replicate). Significances were determined by one-way ANOVA. P < 0.05.
